# Intron-Based Single Transcript Unit CRISPR Systems for Plant Genome Editing

**DOI:** 10.1186/s12284-020-0369-8

**Published:** 2020-02-03

**Authors:** Zhaohui Zhong, Shishi Liu, Xiaopei Liu, Binglin Liu, Xu Tang, Qiurong Ren, Jianping Zhou, Xuelian Zheng, Yiping Qi, Yong Zhang

**Affiliations:** 1 Department of Biotechnology, School of Life Sciences and Technology, Center for Informational Biology, University of Electronic Science and Technology of China, Room 216, Main Building, No. 4, Section 2, North Jianshe Road, Chengdu, 610054 People’s Republic of China; 2 Jiangsu Key Laboratory of Crop Genomics and Molecular Breeding, Agricultural College, Yangzhou University, Yangzhou, 225009 China; 3 Department of Plant Science and Landscape Architecture, University of Maryland, College Park, MD 20742 USA; 4 Institute for Bioscience and Biotechnology Research, University of Maryland, Rockville, MD 20850 USA

**Keywords:** STU CRISPR 3.0, Cas9, Cas12a, iSTU, Rice

## Abstract

**Background:**

Expression of either Cas9 or Cas12a and guide RNAs by a single Polymerase II (Pol II) promoter represents a compact CRISPR expression system and has many advantages for different applications. In order to make this system routine in plant biology, engineering efforts are needed for developing and optimizing such single transcript unit (STU) systems for plant genome editing.

**Results:**

To develop novel intron-based STU (iSTU) CRISPR system (STU CRISPR 3.0), we first evaluated three introns from three plant species for carrying guide RNAs by using an enhanced green fluorescence protein (eGFP) system in rice. After validation of proper intron slicing, we inserted these gRNA-containing introns into the open reading frames (ORFs) of Cas9 and Cas12a for testing their genome editing capability. Different guide RNA processing strategies have been tested for Cas9 and Cas12a. We demonstrated singular genome editing and multiplexed genome editing with these iSTU-Cas9 and iSTU-Cas12a systems.

**Conclusion:**

We developed multiple iSTU-CRISPR/Cas9 and Cas12a systems for plant genome editing. Our results shed light on potential directions for further improvement of the iSTU systems.

## Background

Sequence-specific nucleases (SSNs), such as zinc finger nucleases (ZFNs), TAL effector nucleases (TALENs) and clustered regularly interspaced short palindromic repeats (CRISPR), have promoted plant reverse genetics and breeding in the past decade (Puchta. 2017, Voytas. 2013). DNA targeting by ZFNs and TALENs is protein-based, which requires co-expression of two protein monomers either separately by two Polymerase II (Pol II) promoters or by one Pol II promoter with a ribosome skipping peptide generating two proteins from a single mRNA (Qi et al. 2013, Shan et al. 2013a, Zhang et al. 2013). Thanks to simple RNA-based DNA targeting, CRISPR/Cas9 and Cas12a systems become leading systems for plant genome editing (Zhang et al. 2019). Application of CRISPR technologies in plants often requires the co-expression of the Cas protein and guide RNAs (gRNAs). There are generally four expression strategies for CRISPR/Cas9 or Cas12a (Zhang et al. 2019). The first strategy relies on a mixed dual promoter system in which the Cas gene is expressed by a Pol II promoter and gRNA is expressed by a Pol III promoter like U6 or U3. This system was used when CRISPR/Cas9 was first demonstrated in plants (Jiang et al. 2013, Li et al. 2013, Nekrasov et al. 2013, Shan et al. 2013b, Zhong et al. 2019). The second strategy relies on a dual Pol II promoter system where the Cas gene and the gRNA are separately expressed by Pol II promoters (Cermak et al. 2017, Tang et al. 2017, Zhong et al. 2018). The third strategy is based on a single Pol II bidirectional promoter which expresses the Cas9 gene and the gRNA in opposite directions, and this system was demonstrated in plants very recently (Ren et al. 2019). The fourth strategy expresses the Cas gene and the gRNA in a single transcript unit (STU). The STU system is the most compact expression system, rendering both CRISPR components under coordinated expression by a single Pol II promoter and a single terminator (Tang et al. 2019, Tang et al. 2016). The STU system is very appealing for applications that require inducible or tissue specific expression, as well as for CRISPR based transcriptional regulations in plants (Lowder et al. 2015).

Precise processing of gRNAs is key to the success of a STU CRISPR system. Previously, processing of a single guide RNA (sgRNA) for Cas9 has been demonstrated with a hammer head (HH)-hepatitis delta virus (HDV) dual ribozyme system (Gao & Zhao. 2014), a tRNA system (Xie et al. 2015), or Csy4 which is a sequence-specific RNase (Tsai et al. 2014). Building on these previous studies, the first demonstration of a CRISPR/Cas9 STU system in plants employed HH ribozyme for precise processing of single guide RNAs (sgRNAs) (Tang et al. 2016). More recently, newer versions of STU systems were developed for plant genome editing where many sgRNA processing strategies have been tested, including Csy4 (Tang et al. 2019) and tRNA (Tang et al. 2019, Wang et al. 2018). Strikingly, expressing sgRNAs without any additional processing also worked in rice (Mikami et al. 2017, Wang et al. 2018). In the case of the STU-Cas12a system, CRISPR RNA (crRNA) processing is either based on Cas12a’s self-processing of direct repeat (DR) CRISPR array (Tang et al. 2019) or HH-HDV ribozyme-based processing (Wang et al. 2018). In these STU systems, the gRNA cassettes were positioned at the 3′ end of the Cas9 gene, separated by a Poly (A) sequence or a linker sequence. In both cases, the Cas gene is not immediately terminated by a conventional terminator, which allows for transcription of the gRNA component. Such a design however may have negative effects on Cas mRNA maturation and gRNA stability. Therefore, it is worthwhile pursuing additional STU strategies, such as positioning gRNAs (e.g. sgRNAs for Cas9 or crRNAs for Cas12a) at the 5′ end of the Cas gene or within a Cas open reading frame (ORF). For these strategies to work, it is inevitable to rely on introns for the expression gRNAs, whether gRNAs for Cas9 or crRNA for Cas12a. Only one recent study has explored the use of introns for gRNA expression in plants (Ding et al. 2018). In that study, a gRNA-containing intron was positioned at the 5′ UTR of the Cas gene and the authors successfully demonstrated genome editing in rice with Cas9 and Cas12a (Ding et al. 2018). However, it could be tricky to apply this system into other plants as the modified 5′ UTR intron may not be compatible with the promoter of choice. Since 5′ UTR introns can often affect transcription and translation (Akua & Shaul. 2013, Cenik et al. 2010, Gallegos & Rose. 2017), it adds another layer of complexity when modifying them because the compatibility of the modified 5′ UTR intron with the promoter of choice has to be empirically tested in a case by case scenario.

The majority of plant endogenous genes contain introns that break apart their ORFs. These introns are embedded in gene bodies and provide numerous opportunities to be repurposed to carry gRNA cassettes. Using introns embedded in gene bodies for gRNA expression would allow us to develop reliable STU CRISPR systems that are independent of promoters of choice, thus making the system highly modular and can be readily used with all kinds of promoters in different plant species. Here, we inserted sgRNA or crRNA into such introns and showed these modified introns did not affect splicing, resulting fully functional proteins. We then inserted such gRNA-carrying introns into the ORFs of Cas9 and Cas12a for testing plant genome editing. We compared three introns from three different plant species and multiple gRNA processing mechanisms. Our work not only demonstrated versatile intron-based STU (iSTU) CRISPR systems (STU CRISPR 3.0) for CRISPR/Cas9 and Cas12a applications in rice, a major crop, but also shed light on future improvement and applications of this technology.

## Results

### Evaluation of Three Introns with an eGFP Reporter System in Rice Protoplasts

**Fig. 1:**
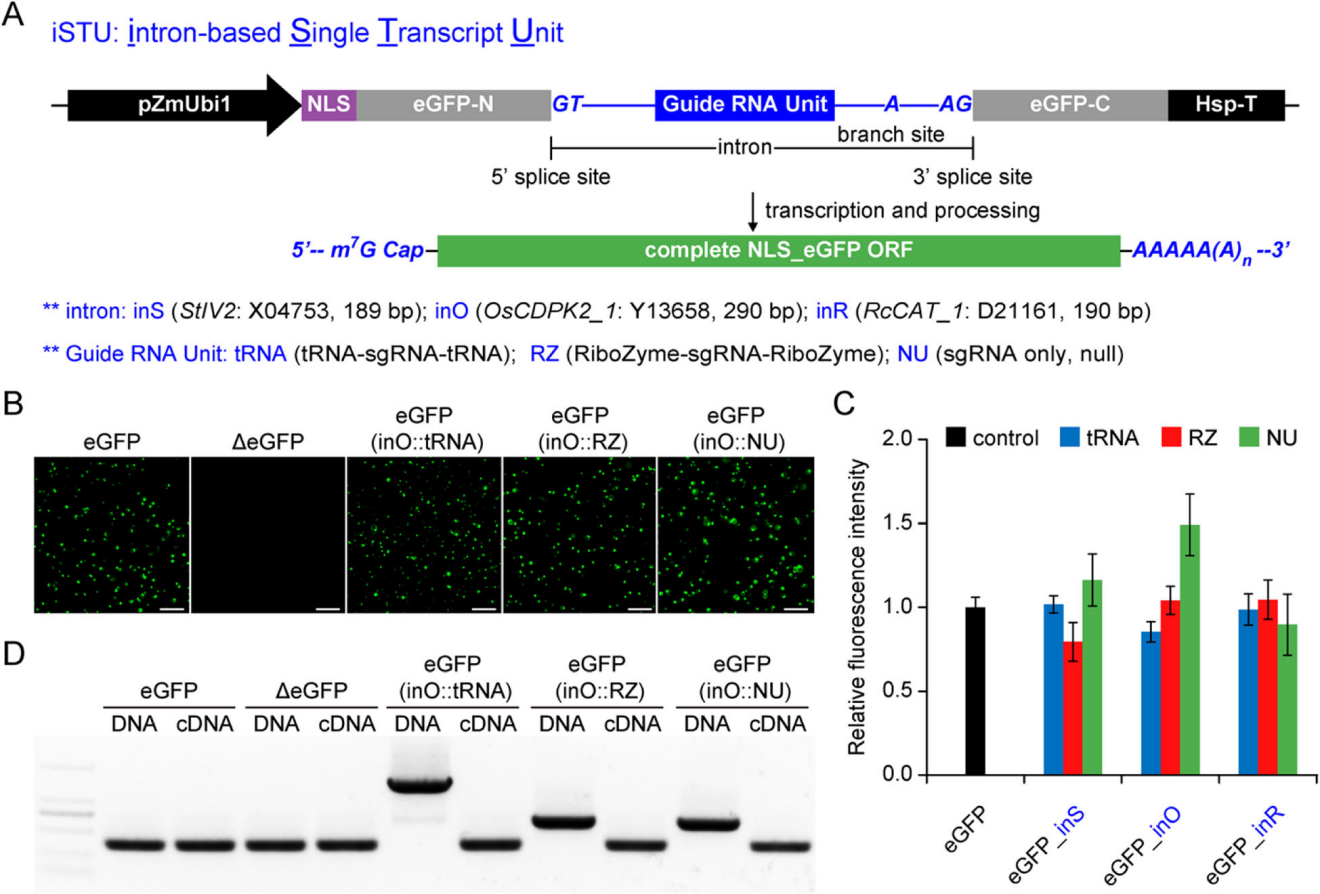
Evaluation of three intron and processing systems with an eGFP reporter in rice protoplasts. **a** Schematic illustration of intron splicing within the eGFP mRNA. Three introns (inS, inO and inR) are shown with accession number and length. Three guide RNA processing units (tRNA, RZ and NU) are compared. **b** Representative images of eGFP expressing protoplasts with the inO intron carrying different guide RNA units. eGFP, enhanced green fluorescence protein; ΔeGFP, eGFP with a frame-shift insertion mutation as a negative control. Scale bar = 100 μm. **c** Quantification of relative fluorescence intensity for eGFP-positive cells among different introns and guide RNA units. Intron-less eGFP was used as a positive control. Data are shown as mean ± s.d. (*n* = 3). The relative fluorescence intensity was quantified by ImageJ. **d** Confirmation of intron splicing by PCR amplification of eGFP from plasmid DNA and Complementary DNA (cDNA)

To develop iSTU systems, we inserted three introns into the OFR of an enhanced green fluorescence protein (eGFP) gene which was drive by the maize ubiquitin 1 (pZmUbi1) promoter (Fig. 1a). The three introns tested were a 189-bp *StIV2* intron (inS) from potato, a 290-bp *OsCDPK2*_1 intron (inO) from rice and a 190-bp *RcCAT*_1 intron (inR) from castor bean. We modified the three introns by inserting tRNA-sgRNA-tRNA cassette (tRNA), HH ribozyme-sgRNA-HH ribozyme (RZ) or null processing sgRNA cassette (NU) in between the 5′ splice site and the branch site (Fig. 1a). A total of nine configurations (three introns by three sgRNA processing mechanisms) were tested. In addition, an intron-less eGFP was included as a positive control and an eGFP with a frame shift mutation (ΔeGFP) was included as a negative control. We transfected rice protoplasts with these 11 T-DNA expression vectors and imaged transfected cells under a fluorescence microscope 2 days after transfection. As expected, majority of protoplasts transfected with the eGFP positive control vector showed GFP signal, while the protoplasts transfected with the ΔeGFP negative control had no GFP signal (Fig. 1b). All three sgRNA processing systems, when positioned in the *OsCDPK2*_1 intron, did not affect intron splicing as most of transfected cells showed GFP signals (Fig. 1b). The same was true for the other two modified introns with three sgRNA processing systems as all six vectors resulted in GFP signal in transfected protoplasts (Additional file 1: Figure S1). We quantified relative fluorescence intensity and found the three modified intron systems (inS, inO and inR) all had equivalent levels of GFP signal to the intro-less vector, regardless the sgRNA processing cassettes (tRNA, RZ or NU) (Fig. 1c). The data suggest that modified intron-containing eGFP vectors achieved similar level of gene expression and efficient splicing of the primary eGFP messenger RNA (mRNA). Focusing on the *OsCDPK2*_1 intron (inO) system, we used PCR and reverse transcription PCR (RT-PCR) to further validate correct splicing products. Indeed, corrected complementary DNA (cDNA) was detected with expected length, shorter than the PCR products with introns amplified from the vector DNA (Fig. 1d). Precise splicing was further validated by Sanger sequencing of cDNA from all nine intron configurations (Additional file 1: Figure S2), suggesting correct joining of two exons after splicing. This phenotypic and molecular evidence collectively suggests that sgRNA-containing introns can be inserted into the ORF of the gene of interest, such as eGFP, without interfering of splicing.

### Comparison of Three sgRNA Processing Systems for iSTU-CRISPR/Cas9 Mediated Genome Editing in Rice Protoplasts

**Fig. 2:**
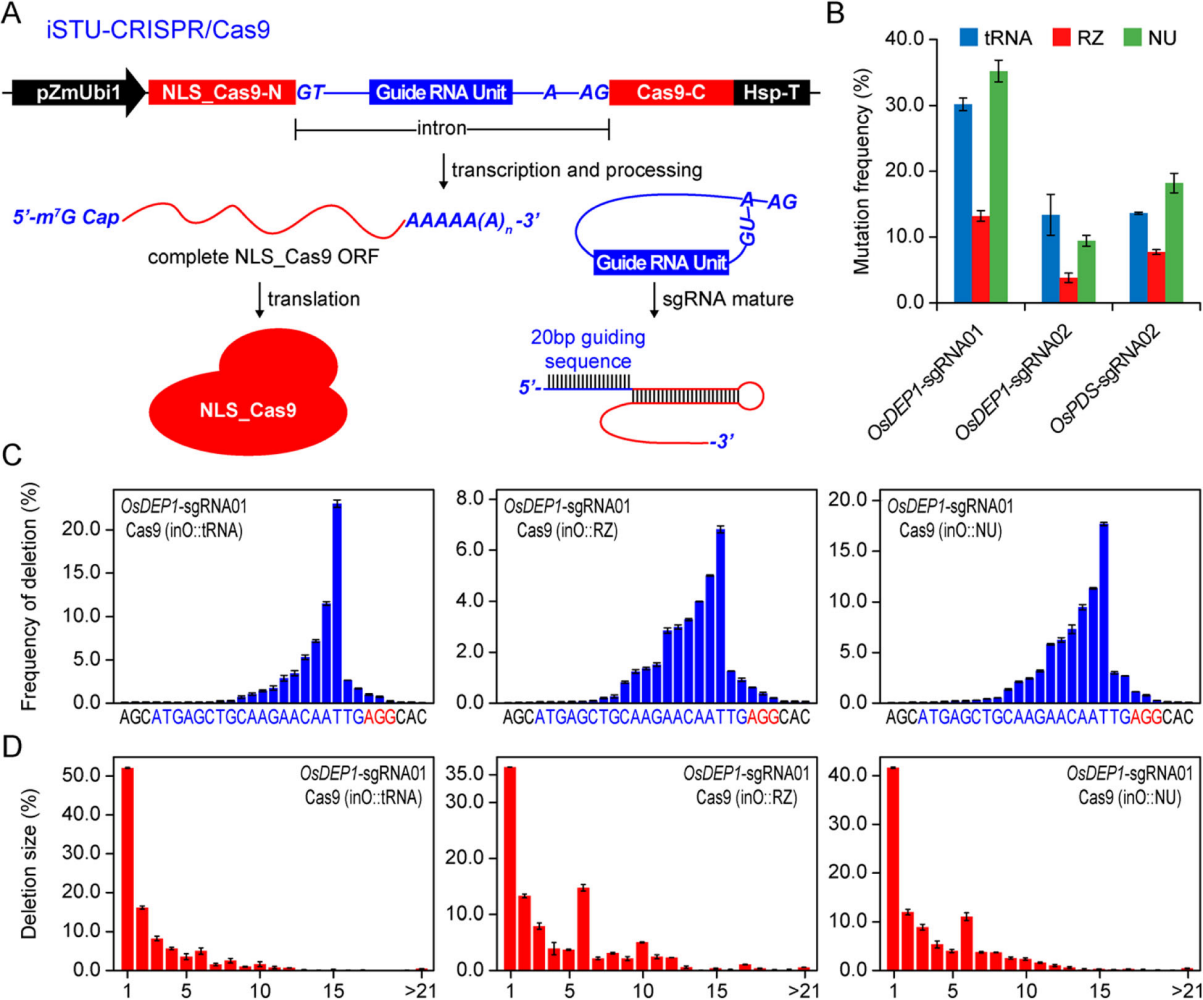
Application of iSTU-CRISPR/Cas9 in rice protoplasts. **a** Schematic illustration of the iSTU-CRISPR/Cas9 system based on Cas9 protein and intron-spliced sgRNA. The lariat structure of intron splicing is shown. GT (U), AG, intron boundaries. **a**, branch site. **b** Quantification of iSTU-CRISPR/Cas9 (inO) induced mutagenesis at *OsDEP1* and *OsPDS* target sites by deep sequencing. The results of three different guide RNA units based on inO were shown. **c, d** The editing profile of iSTU-CRISPR/Cas9 (inO) at *OsDEP1*-sgRNA01 target site. The frequencies of deletion at different positions (**c**) and the total deletion sizes (**d**) were quantified by deep sequencing. Data for ‘B-D’ are shown as mean ± s.d. (*n* = 3)

To determine these spliced introns can carry functional sgRNAs that mediate Cas9 genome editing, we cloned functional protospacer sequences into the sgRNA scaffolds and inserted such modified introns into the ORF of SpCas9 from *Streptococcus pyogenes* (Fig. 2a). It was anticipated that these introns would be properly spliced to result in mature mRNA of Cas9 for protein translation. Also, the spliced sgRNA-containing introns would be properly processed to generate mature sgRNA, and subsequently enable genome editing when the sgRNA form complex with Cas9. We termed this system as intron-based single transcript unit CRISPR/Cas9 (iSTU-CRISPR/Cas9) systems (Fig. 2a). To test iSTU-CRISPR/Cas9, we targeted three independent sites in the rice genome with three sgRNAs, *OsDEP1*-sgRNA01, *OsDEP1*-sgRNA02 and *OsPDS*-sgRNA02. We generated 27 iSTU-CRISPR/Cas9 T-DNA vectors with all possible combination of three intron systems (inS, inO and inR) and three sgRNA processing systems (NU, tRNA and RZ) at three target sites. We transfected these constructs into rice protoplasts. The results based on restriction fragment length polymorphism (RFLP) showed these constructs all worked in generating targeted insertions and deletions (InDels), which destroyed the restriction enzymes sites of *Mun*I, *Hha*I and *Hind*III at the three target sites (Additional file 1: Figure S3). Given that the *OsCDPK2*_1 intron is derived from the rice genome, we decided to subsequently focus on this intron system (inO) in the study. We used next-generation sequencing (NGS) of PCR amplicons to quantify NHEJ mutation frequencies. The results suggested both the tRNA and NU systems led to robust editing, with efficiencies in rice protoplasts over 10% across all three target sites (Fig. 2b). By contrast, the RZ system had relatively poor activity; mutation frequencies were about half compared to the tRNA or the NU system (Fig. 2b).

We next analyzed the NGS data to reveal mutation profiles. Analysis of the results with *OsDEP1*-sgRNA01 showed that the frequencies of deletions at different positions were very similar among all three sgRNA processing systems for the target site of *OsDEP1*-sgRNA01 (Fig. 2c), with 1-bp deletions as the most frequent mutations (Fig. 2d). Analysis of the mutation profiles at *OsDEP1*-sgRNA02 and *OsPDS*-sgRNA02 sites (Additional file 1: Figure S4) further reinforced these findings. In general, the deletion profiles are very similar to our previous reports with the conventional mixed dual promoter Cas9 system (Tang et al. 2018), the single transcript unit systems (Tang et al. 2019, Tang et al. 2016), or the bidirectional promoter systems (Ren et al. 2019). The results suggest that NHEJ outcomes by Cas9 are largely reproducible and independent of the CRISPR/Cas9 expression systems.

**Fig. 3:**
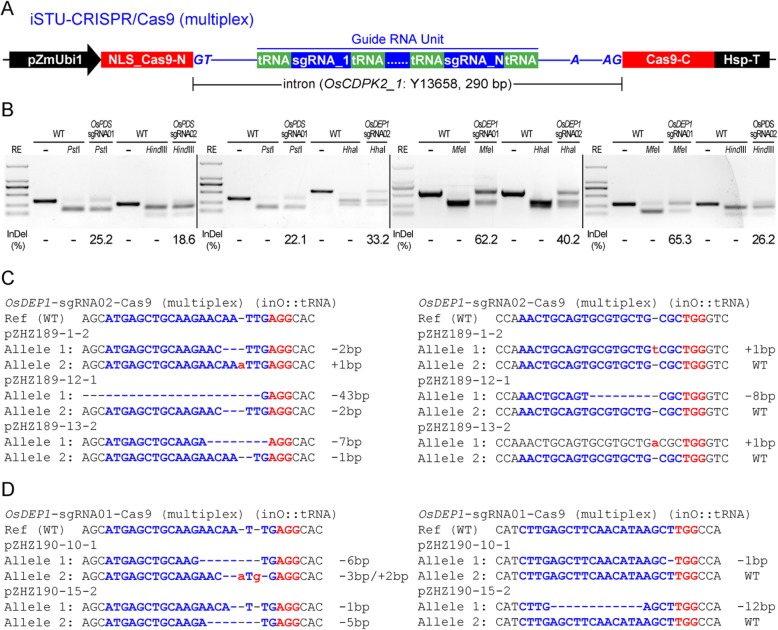
Application of a multiplexed iSTU-CRISPR/Cas9 system in rice. **a** Schematic illustration of a multiplexed iSTU-CRISPR/Cas9 (inO::tRNA) system based on tRNA processing. **b** The RFLP analysis of iSTU-CRISPR/Cas9 (inO::tRNA) multiplexing at *OsPDS* and *OsDEP1* target sites. The insertion and deletion (InDel) percentage was quantified by ImageJ. **c** Sanger sequencing of three T0 lines that carried mutations at both target sites by the inO::tRNA based multiplex system. **d** Sanger sequencing of two T0 lines that carried mutations at both target sites by the inO::tRNA based multiplex system

To investigate whether the iSTU-CRISPR/Cas9 system can be used for multiplexed genome editing, we chose the tRNA system and four genomic sites (*OsPDS*-sgRNA01, *OsPDS*-sgRNA02, *OsDEP1*-sgRNA01 and *OsDEP1*-sgRNA02). We targeted these sites two a time with four combinations: *OsPDS*-sgRNA01 and *OsPDS*-sgRNA02, *OsPDS*-sgRNA01 and *OsDEP1*-sgRNA02, *OsDEP1*-sgRNA01 and *OsDEP1*sgRNA02, and *OsDEP1*-sgRNA01 and *OsPDS*-sgRNA02 (Fig. 3a). The multiplexed T-DNA vectors were generated and transfected into rice protoplasts. Analysis by RFLP showed editing efficiencies at these target sites ranged from 18.6% to 65.3% (Fig. 3b). These data demonstrated multiplexed editing in protoplasts by the inO::tRNA based iSTU-CRISPR/Cas9 system.

### Comparison of iSTU-CRISPR/Cas9 Genome Editing Systems in Stable Transgenic Lines

We next conducted rice stable transgenesis to further compare multiple iSTU-CRISPR/Cas9 genome editing systems. To this end, we transformed rice calli with 12 T-DNA vectors corresponding to three sgRNA processing systems (inO::tRNA, inO::RZ and inO::NU) at four target sites: *OsDEP1*-sgRNA01, *OsDEP1*-sgRNA02, *OsPDS*-sgRNA01 and *OsPDS*-sgRNA02. Up to 50 transgenic T0 lines were obtained for each construct and these T0 lines were genotyped by Sanger sequencing followed by decoding. The results suggested high editing efficiency with *OsDEP1*-sgRNA01 (as high as 90% with the inO::tRNA system) and relatively lower efficiencies at the other three target sites (Table 1), which is consistent with the data from protoplasts (Fig. 2b and Fig. 3). Overall, the inO::tRNA system outperformed the inO::RZ and inO::NU systems. Surprisingly, the inO::RZ system showed equal or better editing efficiencies over the inO::NU system. For example, the inO::tRNA system resulted in editing efficiencies of 71.4% and 84.6% at *OsDEP1*-sgRNA01 and *OsPDS*-sgRNA01 sites, higher than the inO::NU system which only had efficiencies of 44.0% and 27.3% at these two sites, respectively (Table 1). Both monoallelic and biallelic mutants were generated by all three iSTU-Cas9 systems at the target sites (Table 1 and Additional files 1: Figures S5–S7).

**Table 1:** Genome editing in stable transgenic rice lines with intron based Cas9 system

Targeted rice sites	CRISPR scaffold	Tested T0 lines	Mutated T0 lines (number; ratio)	Biallelic mutation lines (number; ratio)	Constructs
*OsDEP1*-sgRNA01	Cas9 (inO::tRNA)	10	9, 90.0%	7, 70.0%	pZHZ159
*OsDEP1*-sgRNA02		12	5, 46.5%	2, 16.7%	pZHZ160
*OsPDS*-sgRNA01		15	9, 60.0%	2, 13.3%	pZHZ161
*OsPDS*-sgRNA02		26	14, 53.8%	6, 23.1%	pZHZ162
*OsDEP1*-sgRNA01	Cas9 (inO::RZ)	14	10, 71.4%	4, 28.6%	pZHZ167
*OsDEP1*-sgRNA02		6	2, 33.3%	1, 16,7%	pZHZ168
*OsPDS*-sgRNA01		18	11, 84.6%	3, 16,7%	pZHZ169
*OsPDS*-sgRNA02		10	1, 10.0%	1, 10.0%	pZHZ170
*OsDEP1*-sgRNA01	Cas9 (inO::NU)	25	11, 44.0%	8, 32.0%	pZHZ171
*OsDEP1*-sgRNA02		12	4, 33.3%	0, 0.0%	pZHZ172
*OsPDS*-sgRNA01		11	3, 27.3%	2, 16.7%	pZHZ173
*OsPDS*-sgRNA02		37	11, 29.7%	1, 9.1%	pZHZ174
*OsDEP1*-sgRNA01	Cas9 (inO::tRNA)	30	17, 58.6%	7, 23.3%	pZHZ189 (Multiplex)
*OsDEP1*-sgRNA02		30	3, 10.0%	0, 0.0%	
*OsDEP1*-sgRNA01	Cas9 (inO::tRNA)	50	30, 71.4%	20, 40.0%	pZHZ190 (Multiplex)
*OsPDS*-sgRNA02		50	2, 4.0%	0, 0.0%	

We also pursued multiplexed editing in rice stable transgenesis with the inO::tRNA based iSTU-CRISPR/Cas9 system. We observed robust editing at the first site, *OsDEP1*-sgRNA01, with editing efficiencies of 58.6% and 71.4% by two constructs respectively. However, we only observed low editing efficiency at the second site either with *OsDEP1*-sgRNA02 (10.0% editing efficiency) or *OsPDS*-sgRNA02 (4.0% editing efficiency) (Table 1). Regardless, we were successful in identifying multiple T0 lines that carried mutations at both target sites (Fig. 3c and d).

### Development of iSTU-CRISPR/Cas12a Systems in Genome Editing in Rice Protoplasts

**Fig. 4:**
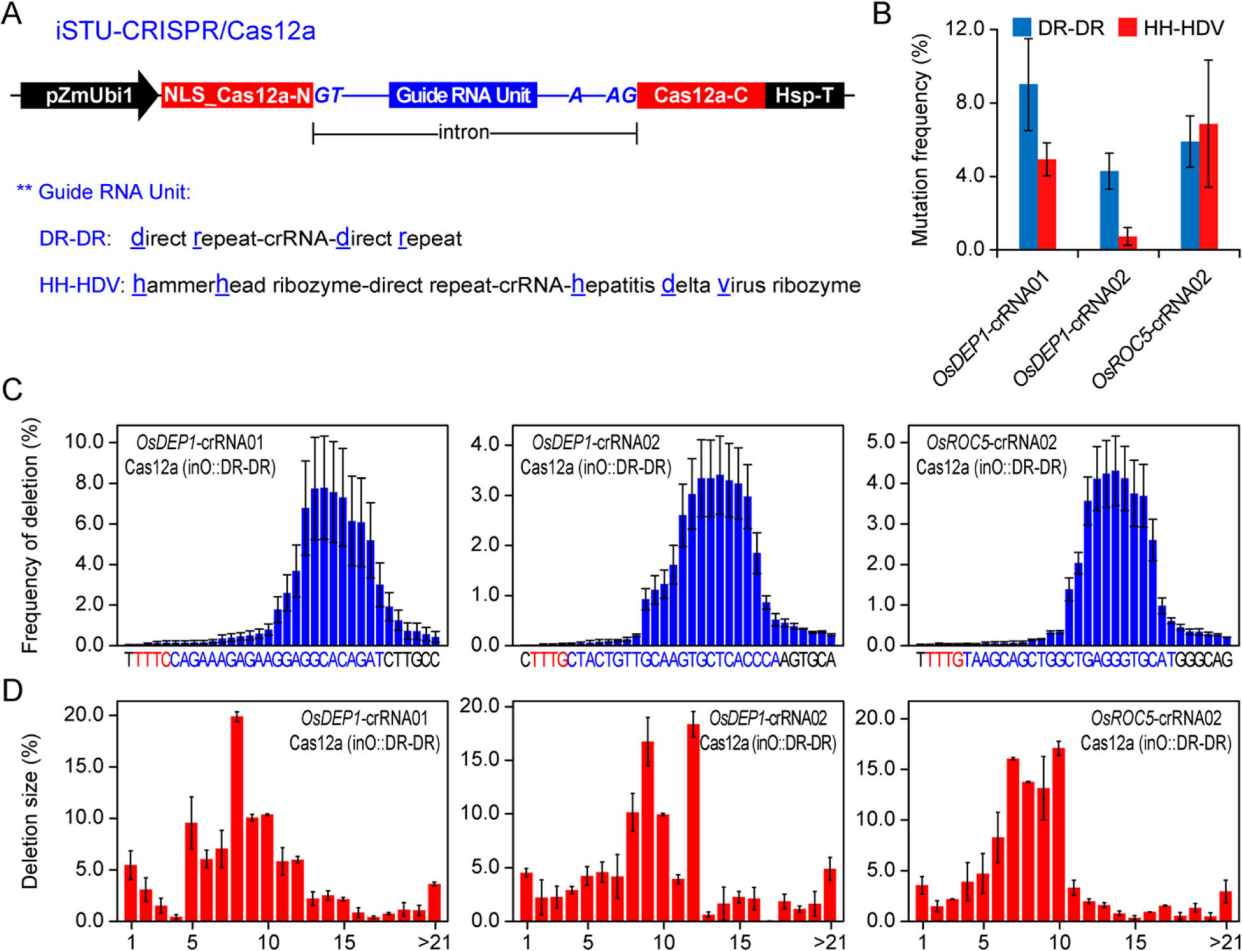
Application of iSTU-CRISPR/Cas12a in rice protoplasts. **a** Schematic illustration of the iSTU-CRISPR/Cas12a expression system. The two different guide RNA processing units, DR-DR and HH-HDV, are shown. **b** Quantification of iSTU-CRISPR/Cas12a (inO) induced mutagenesis at *OsDEP1* and *OsROC5* target sites by deep sequencing. (C-D) The editing profile of iSTU-CRISPR/Cas12a (inO) based the DR-DR unit. Deletion frequencies of different positions (**c**) and the total deletion size (**d**) were quantified by deep sequencing. Data for ‘B-D’ are shown as mean ± s.d. (*n* = 3)

We reasoned that our intron systems could also be used to express crRNAs for CRISPR-Cas12a mediated genome editing. There are two popular systems for processing crRNAs to maturity in plants, one based on self-cleavage of direct repeat (DR) by Cas12a’s intrinsic RNase activity (Fonfara et al. 2016, Zetsche et al. 2017) and the other based on a dual HH-HDV ribozyme system (Tang et al. 2017). We positioned the DR-crRNA-DR or the HH-crRNA-HDV cassette in between the 5′ splice site and the branch site of the *OsCDPK2*_1 intron, and the modified intron with a crRNA unit was inserted into the ORF of a rice codon-optimized LbCas12a from *Lachnospiraceae* bacterium ND2006 (Tang et al. 2017) (Fig. 4a). To compare these two iSTU-CRISPR/Cas12a systems, we targeted three rice genomic sites with *OsDEP1*-crRNA01, *OsDEP1*-crRNA02 and *OsROC5*-crRNA02. The resulting six expression vectors were used for rice protoplast transfection. Targeted mutations at these three sites were evaluated by RFLP, which showed detectable mutations at all three target sites (Additional file 1: Figure S8). We then used NGS to further quantify mutation frequencies at the target sites. The DR-DR system showed higher editing efficiencies than the HH-HDV system at *OsDEP1*-crRNA01 and *OsDEP1*-crRNA02 sites, while both systems showed equivalent editing efficiencies at *OsROC5*-crRNA02 site (Fig. 4b). These data indicate that the DR-DR system is more robust than the HH-HDV system when coupled with the intron expression system.

We analyzed the NGS data for deletion profiles by the iSTU-CRISPR/Cas12a systems. The DR-DR system resulted in deletions that mostly affected the nucleotides close to the cleavage sites and the deletions were much larger than Cas9 induced deletions (Fig. 4c and d). The same deletion profiles were revealed by the HH-HDV system (Additional file 1: Figure S9). These data reinforce our previous conclusions regarding Cas12a induced large deletions in plants (Malzahn et al. 2019, Tang et al. 2018, Tang et al. 2017, Tang et al. 2019, Zhong et al. 2018), which support that overall mutation profiles by Cas12a are also reproducible within a large cell population and such profiles are not affected by the expression systems.

**Fig. 5:**
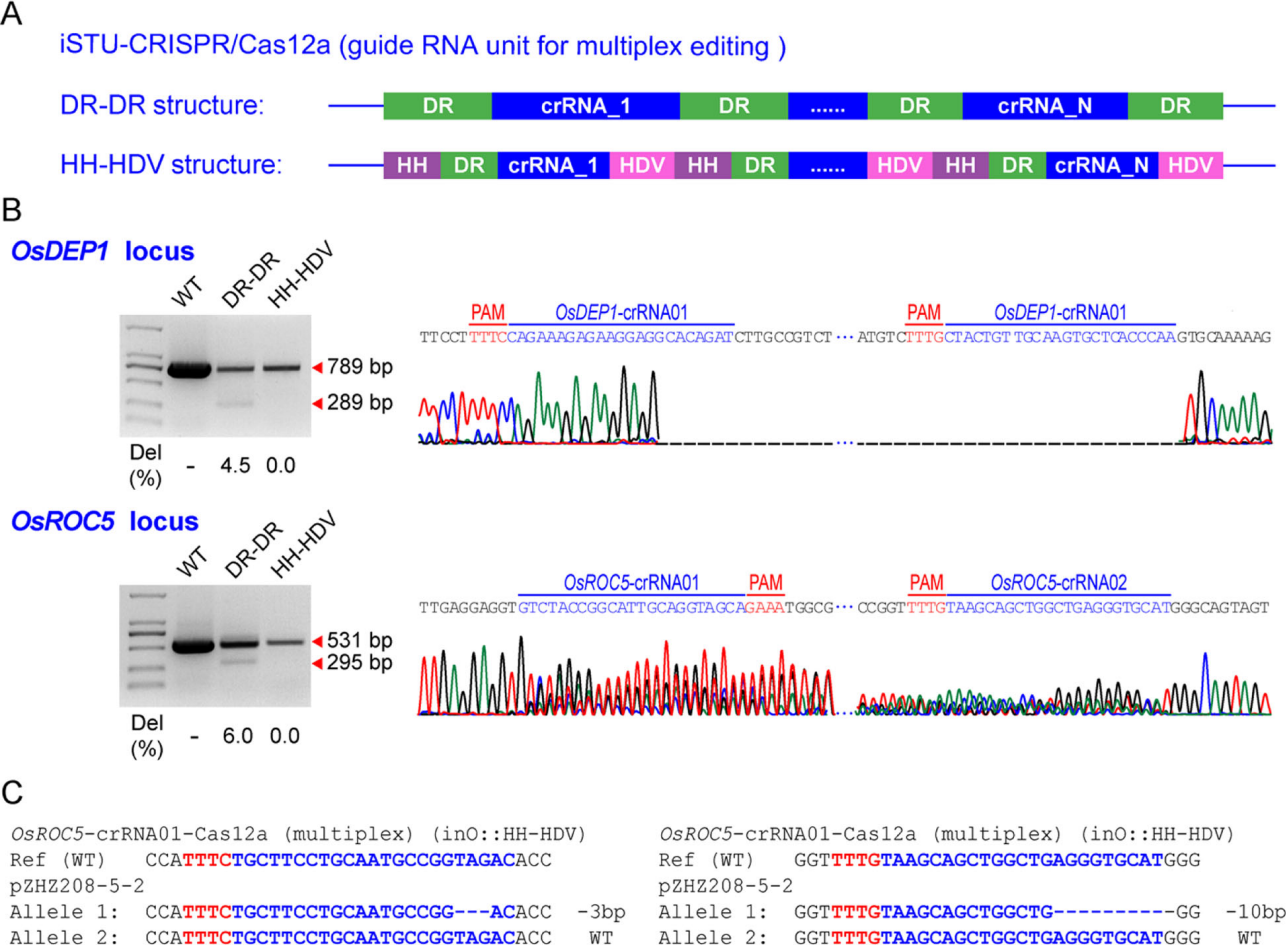
Application of a multiplexed iSTU-CRISPR/Cas12a system in rice. **a** Schematic illustration of the multiplexed iSTU-CRISPR/Cas12a expression system. **b** Gel electrophoresis and Sanger sequencing results of iSTU-CRISPR/Cas12a (inO) induced gene deletions at *OsDEP1* and *OsROC5* loci. Left, detection of targeted deletions by PCR. Deletion percentage was quantified by ImageJ. Right, Sanger sequencing results that further validated the deletions. **c** Sanger sequencing of one T0 line that carried mutations at both target sites by multiplexed genome editing

We next used DR-DR based CRISPR array and an HH-HDV array to multiplex two crRNAs with the iSTU-CRISPR/Cas12a system (Fig. 5a). We simultaneously expressed two crRNAs for deletion of a DNA fragment of 289 bp in *OsDEP1* and used another two crRNAs for deletion of a DNA fragment of 295 bp in *OsROC5*. Two multiplexed Cas12a constructs were generated for rice protoplast transfection. For the DR-DR samples, we detected chromosomal deletions by PCR at both genes with frequencies of 4.5% and 6%, respectively, and the large deletions were further validated by Sanger sequencing (Fig. 5b). However, we could not detect deletion events at either gene with PCR for the HH-HDV system (Fig. 5b). The data suggest the DR-DR based CRISPR array, not the HH-HDV array, is more suitable for intron-based processing that enables Cas12a mediated multiplexed genome editing. This observation is consistent with our earlier findings regarding iSTU-CRISPR/Cas12a system with singular crRNAs (Fig. 4b).

### Evaluation of iSTU-CRISPR/Cas12a Systems for Genome Editing in Stable Transgenic Lines

To assess whether we can obtain mutants with the iSTU-CRISPR/Cas12a systems, we compared the DR-DR and HH-HDV systems in stable rice transformants (Table 2). With *OsDEP1*-crRNA01, the DR-DR system generated 7 mutants out of 15 T0 lines (46.7%) and the HH-HDR only generated one mutant out of 19 T0 lines (5.3%). With *OsROC5*-crRNA01, the DR-DR system generated 2 mutants out of 22 T0 lines (9.1%) and the HH-HDV failed to generate any mutant out of 21 T0 lines. These data suggest the DR-DR system is more robust than the HH-HDV system for iSTU-CRISPR/Cas12a mediated targeted mutagenesis in stable rice lines, consistent with our observations in rice protoplasts.

**Table 2:** Genome editing in stable transgenic rice lines with intron based Cas12a system

Targeted rice sites	CRISPR scaffold	Tested T0 lines	Mutated T0 lines (number; ratio)	Biallelic mutation lines (number; ratio)	Constructs
*OsDEP1*-crRNA01	Cas12a (inO::DR-DR)	15	7, 46,7%	0, 0.0%	pZHZ144
*OsROC5*-crRNA-01		22	2, 9.1%	0, 0.0%	pZHZ146
*OsDEP1*-crRNA01	Cas12a (inO:: HH-HDV)	19	1, 5.3%	0, 0.0%	pZHZ148
*OsROC5*-crRNA-01		21	0, 0.0%	0, 0.0%	pZHZ150
*OsROC5*-crRNA01	Cas12a (inO:: HH-HDV)	22	1, 4.5%	0, 0.0%	pZHZ208 (Multiplex)
*OsROC5*-crRNA02		22	3, 13.7%	0, 0.0%	

In rice protoplasts, the HH-HDV based multiplex system did not generate detectable large deletions at both target genes when assayed by PCR (Fig. 5b), which may be largely due to its low editing efficacy. This system however may be able to introduce targeted mutations at individual sites. We investigated this possibility with rice stable transgenesis using the multiplexed HH-HDV construct targeting *OsROC5*. Among 22 T0 lines assayed, one line showed mutation by *OsROC5*-crRNA01 and three lines had mutations by *OsROC5*-crRNA02, resulting mutation frequencies of 4.5% and 13.7%, respectively (Table 2). Notably, one line (pZHZ208–5-2) had mutations in both target genes (Fig. 5c). The data suggest the multiplexed HH-HDV array, when configured into the iSTU system, can generated rice mutants at individual target sites, albeit at a low efficiency.

## Discussion

Since many plant genes contain introns, it is intriguing to configure a transgene such as Cas9 or Cas12a that contains introns. First, it is well known that some introns can boost gene expression (Jeong et al. 2006). The plant codon optimized Cas9 (pcoCas9) contains an intron (Li et al. 2013), which may prevent the Cas9 transgene from gene silencing across multiple generations. We hence adopted pcoCas9 for engineering synthetic transcriptional repressors and activators (Lowder et al. 2015, Lowder et al. 2018). Second, adding introns to the transgene can abolish potential toxicity in bacteria (e.g. *E. coli*) during the molecular cloning process. For example, we previously introduced an intron into the ORF of TAL effector nuclease (TALEN) to avoid toxicity in *E. coli* and *Agrobacterium* (Christian et al. 2013). Third, insertion of an gRNA-containing intron to the ORF of Cas9 or Cas12a represents a more authentic gene structure for expression of both the Cas gene and the gRNA in a single mRNA. It is thus imperative to the development of novel intron-based STU CRISPR genome editing systems. With appropriate intron choices and proper positioning in the Cas gene, one would configure a novel STU CRISPR-Cas system that mimics an endogenous gene structure. Previously, Ding et al. explored a 5′ UTR intron to express sgRNA or crRNA for Cas9 or Cas12a mediated genome editing in rice (Ding et al. 2018). Compared to the 5′ UTR, there are numerous positions in the ORFs of Cas9 and Cas12a for intron insertions. The only previous study that employed an intron to express an sgRNA within a Cas9 for plant genome editing was done in *Chlamydomonas reinhardtii*, which showed very low editing efficiency likely due to toxicity of Cas9 in this green algae (Jiang & Weeks 2017). In this study, we explored multiple iSTU systems where gRNA-containing introns were inserted in the middle of a Cas9 or Cas12a ORF. We tested three introns from three plant species, including an *StIV2* intron from potato, an *OsCDPK2*_1 intron from rice and a *RcCAT*_1 intron from castor bean. Using an eGFP reporter system, we first demonstrated these introns, when carrying gRNA units, could be properly spliced in rice. We then applied these introns to carry functional sgRNA or crRNA cassettes and successfully showed genome editing by either Cas9 or Cas12a, in both protoplasts and stable transgenic rice lines. Our success suggests many different introns may be amendable to carry gRNAs for CRISPR genome editing in plants.

For detailed assessment of our iSTU systems, we later focused on the *OsCDPK2*_1 intron (e.g. inO) as it is an endogenous intron from the rice genome. With the inO-Cas9 system, we compared insertion of sgRNAs with and without added processing mechanisms. We found tRNA based processing is most robust for genome editing in both protoplasts and stable transgenic lines. The processing based on a single HH ribozyme is less efficient. These observations are consistent with our recent findings when developing CRISPR-STU2.0 systems (Tang et al. 2019). Interestingly, we also found the iSTU system without any added processing mechanism also worked, which showed equivalent editing efficiencies to the HH ribozyme systems. This result is consistent with previous findings (Mikami et al. 2017, Wang et al. 2018), indicating possible sgRNA maturation based on an unknown RNase in rice. With the inO-Cas12a system, we compared two crRNA processing mechanisms, DR-DR and HH-HDV. The DR-DR inO-Cas12a system showed much better results than the one based on HH-HDV. Our most efficient Cas12a system is a dual Pol II promoter system which relies on HH-HDV for crRNA processing (Tang et al. 2017, Zhong et al. 2018). The contrasting efficiencies of the HH-HDV processing strategies in different expression systems suggest the self-cleavage feature of HH and HDV ribozymes may not be compatible with the intron system. Although we did not find abnormal splicing with an HH-ribozyme carrying intron in the eGFP reporter system, it is possible the instant self-cleavage by HH and HDV ribozymes negatively affected intron splicing or crRNA maturation, which can explain the observed low editing efficiency with the HH-HDV based iSTU-Cas12a system.

The overall genome editing efficiency of our iSTU systems, either with Cas9 or Cas12a, is similar in some degree to 5′ UTR intron systems previously reported (Ding et al. 2018). Compared to the 5′ UTR intron systems, our iSTU systems are promoter-independent and should be readily applicable to other plant species. At this point, these intron-based CRISPR systems seem sub-optimal in terms of genome editing efficiency when compared to other STU systems where gRNA cassettes were positioned at the 3′ end (Tang et al. 2019, Tang et al. 2016, Wang et al. 2018). We noted our iSTU systems became less efficient when more than one gRNA was inserted into the intron, making our systems less robust for multiplexed genome editing. This may be explained by the use of relatively small introns as all three introns we used were under 300 bp. It is likely that insertion of multiple gRNA cassettes into these small introns significantly affect the intron structure. It is thus worthwhile to test larger introns in future investigations which may improve the overall genome editing efficiency of the iSTU systems. Alternatively, it may be more effective to use multiple introns to individually carry each gRNA cassette for developing multiplexed iSTU systems. Based on our data with singular gRNA and multiple gRNAs, it appears such a multi-intron strategy may work better and hence is worth future exploration.

While splicing sites can be designed and validated relatively easily, the fate of the introns after splicing is largely unknown. It is also unknown whether insertion of gRNA cassettes into an intron will affect its turnover rate. Given Cas9 and Cas12a each has an ORF well over 3 kb, there are many positions within the ORF for intron insertion. In this study, we only arbitrarily chose one site in Cas9 or Cas12a for intron insertion. It is hard to believe the position that we chose is the ideal position. While many intron insertion sites may be tested, we believe the choice of introns and the choice of gRNA insertion site are both key to enhanced editing efficiency. While the focus of this study is to develop iSTU systems for plant genome editing, we envision such iSTU systems may be useful for CRISPR based transcriptional regulation. The iSTU systems may also allow for indirect measurement of splicing efficiency for certain introns as well as for understanding the fate of introns after splicing.

## Conclusion

With rice as a test platform, we developed multiple iSTU systems (STU CRISPR 3.0) for Cas9 and Cas12a mediated genome editing in plants. While the genome editing efficiency is still sub-optimal, this proof-of-concept study has shed light on improved iSTU systems in plants.

## Materials and Methods

### Vector Construction

The eGFP-intron expression, iSTU-CRISPR/Cas9 and iSTU-CRISPR/Cas12a backbone vectors in this study were constructed by Gibson Assembly® Cloning Kit from New England Biolabs (Ipswich, MA, USA). Plasmid was isolated using Axygen Mini Plasmid Kit (Corning, NY, USA). Gel extraction was conducted using AxyPrep DNA Gel Extraction Kit (Corning, NY, USA). Oligonucleotides were synthesized by Sangon Biotech (Shanghai, CHN). The DNA fragments were synthesized by IDT (Integrated DNA Technologies, IA, USA). The fast digest enzymes were purchased from Thermo Fisher Scientific (Waltham, MA, USA). All other reagents used here were purchased from New England Biolabs (Ipswich, MA, USA). All the oligos were summarized in Additional file 2: Table S1.

To generate the eGFP-intron expression backbone, three introns with three guide RNA unit structures (NU, tRNA, RZ) were synthesized. To generate pZHZ113 for the eGFP expression, eGFP was amplified with primers ZHZ113-A and ZHZ113-B from pZHY981. The tHSP part was amplified from pZHY988 with primers ZHZ113-C and ZHZ113-D. Both PCR fragments were assembled with *Sda*I and *Hind*III digested pZHZ127 plasmid by Gibson Assembly. The final vector was sequenced by ZY065-RB and ZmUbi-Seq for confirmation. To generate pZHZ114 for the ΔeGFP expression, the eGFP fragment 1 with *Bsa*I insertion was PCR amplified with primers ZHZ113-A and ZHZ113-E from pZHZ113; the eGFP fragment 2 with *Bsa*I insertion and tHSP part were PCR amplified with ZHZ113-F and ZHZ113-D from pZHZ113; the three parts were fused with primers ZHZ113-A and ZHZ113-D by fusion PCR. The final vector was generated by Gibson Assembly cloned into *Sda*I and *Hind*III digested pZHZ113 plasmid and sequenced by ZY065-RB and ZmUbi-Seq for confirmation. The resulting ΔeGFP was frame-shifted with a 16 bp insertion. To generate pZHZ118-pZHZ126 eGFP expression vectors with different introns and different guide RNA unit structures, nine DNA fragments were synthesized by IDT. The diluted fragments were cloned into *Bsa*I digested pZHZ114 plasmid by Gibson Assembly. The final vector was sequenced by ZY065-RB and ZmUbi-Seq for confirmation. Notably, the tRNA based guide RNA structure contained a *ccdB* gene insertion.

To generate iSTU-Cas9 expression vector with different intron and different guide RNA unit insertions, the Cas9 fragment 1 with *Bsa*I insertion was PCR amplified with primers ZHZ113-A and ZHZ112-B. The Cas9 fragment 2 and tHSP part were amplified with primers ZHZ112-C and ZHZ113-D. These two fragments were fused together by fusion PCR with primers ZHZ113-A and ZHZ113-D. The fused fragment was cloned into pZHZ127 by Gibson Assembly to make pZHZ128. To generate iSTU-CRISPR/Cas9 backbone vectors, pGEL077 to pGEL086, nine fragments were amplified from pZHZ118 to pZHZ126 with specific primer sets (Additional file 2: Table S1), and then cloned into *Bsa*I digested pZHZ128 plasmid by Gibson Assembly. The vector was confirmed by Sanger sequencing using primers dCas9-F and dCas9-R.

To generate iSTU-CRSPR/Cas12a backbone vector pGEL087, oligos ZHZ141-F and ZHZ141-R were annealed and ligated into *Spe*I and *Asc*I sites of pTX377 to remove the crRNA expression cassette, resulting in pGEL087-Step1. The LbCas12a part I was PCR amplified from pTX377 with primers ZHZ141-F1 and ZHZ141-R1. The LbCas12a part II was amplified from pTX377 with primers ZHZ141-F2 and ZHZ141-R2. The two parts were fused via PCR with primers ZHZ141-F1 and ZHZ141-R2, and the resulting PCR fragment was cloned into *Bgl*II and *Sph*I sites of pGEL087-Step1 plasmid. To generate iSTU-CRISPR/Cas12a vector, pGEL088 and pGEL089, the synthesized fragments were cloned into *Bsa*I digested pGEL087 by Gibson Assembly.

To generate the T-DNA vector, the oligos were synthesized based on the target sites selected. The oligo pair for each guide RNA was annealed and inserted into *Bsa*I sites of the backbone vectors by Golden Gate cloning according our published protocol (Lowder et al. 2015). To generate iSTU-CRISPR/Cas9 and iSTU-CRISPR/Cas12a multiplex T-DNA vectors, the synthesized fragments were cloned into corresponding backbones by Golden Gate cloning. These T-DNA expression vectors (Additional file 3: Table S2) were confirmed by RFLP and Sanger Sequencing.

### Plant Material and Growth Condition

The *Japonica* cultivar Nipponbare was used in this study. For protoplast preparation, the sterilized seeds were germinated in the 1/2 MS solid medium for 11 days in dark chamber at 28 °C. For the rice stable transformation, the sterilized seeds were germinated in the N6-D solid medium to induce the callus for 7 days in light at 32 °C.

### Rice Protoplast Preparation, Transformation and Analysis

The rice protoplast extraction and transformation method was performed according to our previously published protocol (Tang et al. 2016, Zhong et al. 2018). Leaves of rice seedlings were cut into 0.5–1.0 mm strips, and transferred into the enzyme solution followed by vacuum-infiltration for 30 min. The mixture was gently agitated at 70–80 rpm for 8 h at 25 °C in the dark. The digestion mixture was filtered by a 40 μm cell strainer. After washing the protoplasts two times with W5 washing buffer, cell counts of protoplasts was done using a hemacytometer under a microscope and the final protoplast concentration was adjust to 2 × 10^6^ per milliliter. For protoplast transformation, 30 μL plasmid DNA (1 μg/ μL; prepared by Axygen midiprep kit) was used for transformation of 200 μL protoplasts by gently mixing with 230 μL 40% PEG transformation buffer. After incubating 30 min in the dark, the reaction was terminated by adding 1 mL W5 washing buffer. The protoplasts were centrifuged at 250×g and transferred into a 12-well culture plate at 32 °C in the dark. After 2 days’ incubation, images of the cells were taken by a fluorescence microscope with a GFP filter or DNA was extracted from the cells using the CTAB method (Murray & Thompson. 1980). The RFLP method was used to detect NHEJ mutations induced by Cas9 or Cas12a at the target sites. The PCR was performed using Tsingke Golden Mix (Tsingke, Beijing) with specific primers (see Additional file 2: Table S1). The PCR products were digested overnight and resolved by electrophoresis in 1% TAE agarose gels.

### Rice Stable Transformation and Genotyping

The Agrobacterium-mediated rice transformation was performed as the protocol we published before (Zhou et al. 2017, Zhou et al. 2019). The calli for stable transformation were cultured in the chamber for 7 days at 32 °C under light. The binary T-DNA vectors were transferred into *Agrobacterium tumefaciens* strain EHA105. The transformed Agrobacterium EHA105 cells were cultured in liquid LB medium at 28 °C for 2 days. The cells were then collected and resuspended in liquid AAM-AS medium (OD600 = 0.1) containing 100 μM acetosyringone. After co-cultivation of Agrobacteria and calli for 3 days, the calli were washed with sterilized water and transferred to N6-S medium containing 400 mg/L Carbenicillin and 50 mg/L Hygromycin for 2 weeks. Then they were transferred to REIII medium with 400 mg/L Carbenicillin and 50 mg/L Hygromycin cultured for 2 weeks, and the resistant calli were later transferred to fresh REIII medium for every 2 weeks until the regeneration of T0 plants. To genotype T0 plants, the SSCP method (Zheng et al. 2016) was used for a first round of screen, followed by Sanger sequencing of positive lines.

### The NGS Sample Preparation, Detection and Analysis

Next-generation sequencing (NGS) of PCR amplicons was also used for detection and quantification of mutations present in protoplast samples. With the protoplast DNA as template, the DNA sequence flanking each target site was amplified by using Tsingke Golden Mix Kit (Tsingke, Beijing) and the barcodes were added to the end of the primers. The PCR products were resolved by electrophoresis in 1% agarose gel for checking purity and concentration. The PCR product were sent to the Novogene and sequenced by using an Illumina Hiseq 2500 platform. The clean data were analysis by CRISPRMatch (You et al. 2018).

## Supplementary information

**Additional file 1: Figure S1.** eGFP expression of inS and inR intron splicing systems in rice protoplasts. Three different guide RNA units were tested, and all showed GFP signals, indicating of correct splicing. Scale bar = 100 μm. **Figure S2.** Sanger sequencing confirmation of inS, inO and inR intron-based slicing of eGFP mRNA. The cDNA of three different intron splicing systems was used for PCR amplification with a primer set flanking the introns. All sanger sequencing results showed correct intron splicing compared with intron-less eGFP. The splicing site was indicated by a dotted red line. **Figure S3.** RFLP based detection of targeted mutagenesis by iSTU-CRISPR/Cas9 in rice protoplasts. Three introns (inS, inO and inR) with three different guide RNA units were tested at *OsDEP1* and *OsPDS* target sites. The restriction enzymes used for RFLP are shown. Uncut bands are indicative of induced mutations by Cas9. **Figure S4.** Editing profiles of iSTU-CRISPR/Cas9 (inO) at two additional target sites in rice protoplasts. The editing profiles of iSTU-CRISPR/Cas9 (inO) at *OsDEP1*-sgRNA02 and *OsPDS*-sgRNA02 target sites. Deletion frequencies at different positions (A) and frequencies of deletion sizes (B) were quantified by deep sequencing. Data are shown as mean ± s.d. (*n* = 3). **Figure S5.** Examples of T0 rice mutants generated by iSTU-CRISPR/Cas9 (inO::tRNA) at four target sites. **Figure S6.** Examples of T0 rice mutants generated by iSTU-CRISPR/Cas9 (inO::RZ) at four target sites. **Figure S7.** Examples of T0 rice mutants generated by iSTU-CRISPR/Cas9 (inO::NU) at four target sites. **Figure S8.** RFLP analysis of iSTU-CRISPR/Cas12a (inO) in rice protoplasts. (a). RFLP analysis of two iSTU-CRISPR/Cas12a systems, inO::DR-DR and inO::HH-HDV. Three target sites were tested. (b). RFLP analysis of two multiplexed iSTU-CRISPR/Cas12a systems, inO::DR-DR and inO::HH-HDV. *OsDEP1* and *OsROC5* locus were tested with each targeted by two crRNAs. ‘A-B’, the restriction enzymes are shown and the InDel percentages were quantified by ImageJ. **Figure S9.** Editing profiles of three target sites by iSTU-CRISPR/Cas12a (inO) HH-HDV system in rice protoplasts. The editing profiles of iSTU-CRISPR/Cas12a (inO) HH-HDV system at *OsDEP1*-crRNA01, *OsDEP1*-crRNA02 and *OsROC5*-crRNA02 target sites. Deletion frequencies of different positions (A) and frequencies of deletion sizes (B) were quantified by deep sequencing. Data are shown as mean ± s.d. (*n* = 3). **Figure S10.** Examples of T0 rice mutants generated by the iSTU-CRISPR/Cas12a (inO) systems.

**Additional file 2: Table S1.** Oligos used in this study.

**Additional file 3: Table S2.** T-DNA constructs used in this study.

## Data Availability

All data generated were presented in the article and included in the Additional files. The NGS data have been deposited to the Sequence Read Archive in National Center for Biotechnology Information (NCBI) under the accession number PRJNA577195.

## Abbreviations

CRISPR/Cas9 or 12a: Clustered regularly interspaced short palindromic repeats/CRISPR-associated protein-9 or 12a
iSTU: intron-based Single transcript unit
RFLP: Restriction fragment length polymorphism
