# Supplementary material for: Intron-Based Single Transcript Unit CRISPR Systems for Plant Genome Editing
Source: Rice (N Y). 2020 Feb 3;13:8. doi: 10.1186/s12284-020-0369-8 (PMC6997322; doi:10.1186/s12284-020-0369-8)
Supplement: Supplementary file 1 — Additional file 1: Figure S1. eGFP expression of inS and inR intron splicing systems in rice protoplasts. Three different guide RNA units were tested, and all showed GFP signals, indicating of correct splicing. Scale bar = 100 μm. Figure S2. Sanger sequencing confirmation of inS, inO and inR intron-based slicing of eGFP mRNA. The cDNA of three different intron splicing systems was used for PCR amplification with a primer set flanking the introns. All sanger sequencing results showed correct intron splicing compared with intron-less eGFP. The splicing site was indicated by a dotted red line. Figure S3. RFLP based detection of targeted mutagenesis by iSTU-CRISPR/Cas9 in rice protoplasts. Three introns (inS, inO and inR) with three different guide RNA units were tested at OsDEP1 and OsPDS target sites. The restriction enzymes used for RFLP are shown. Uncut bands are indicative of induced mutations by Cas9. Figure S4. Editing profiles of iSTU-CRISPR/Cas9 (inO) at two additional target sites in rice protoplasts. The editing profiles of iSTU-CRISPR/Cas9 (inO) at OsDEP1-sgRNA02 and OsPDS-sgRNA02 target sites. Deletion frequencies at different positions (A) and frequencies of deletion sizes (B) were quantified by deep sequencing. Data are shown as mean ± s.d. (n = 3). Figure S5. Examples of T0 rice mutants generated by iSTU-CRISPR/Cas9 (inO::tRNA) at four target sites. Figure S6. Examples of T0 rice mutants generated by iSTU-CRISPR/Cas9 (inO::RZ) at four target sites. Figure S7. Examples of T0 rice mutants generated by iSTU-CRISPR/Cas9 (inO::NU) at four target sites. Figure S8. RFLP analysis of iSTU-CRISPR/Cas12a (inO) in rice protoplasts. (a). RFLP analysis of two iSTU-CRISPR/Cas12a systems, inO::DR-DR and inO::HH-HDV. Three target sites were tested. (b). RFLP analysis of two multiplexed iSTU-CRISPR/Cas12a systems, inO::DR-DR and inO::HH-HDV. OsDEP1 and OsROC5 locus were tested with each targeted by two crRNAs. ‘A-B’, the restriction enzymes are shown and the InDel percent [file 12284_2020_369_MOESM1_ESM.pptx]

## Slide 1
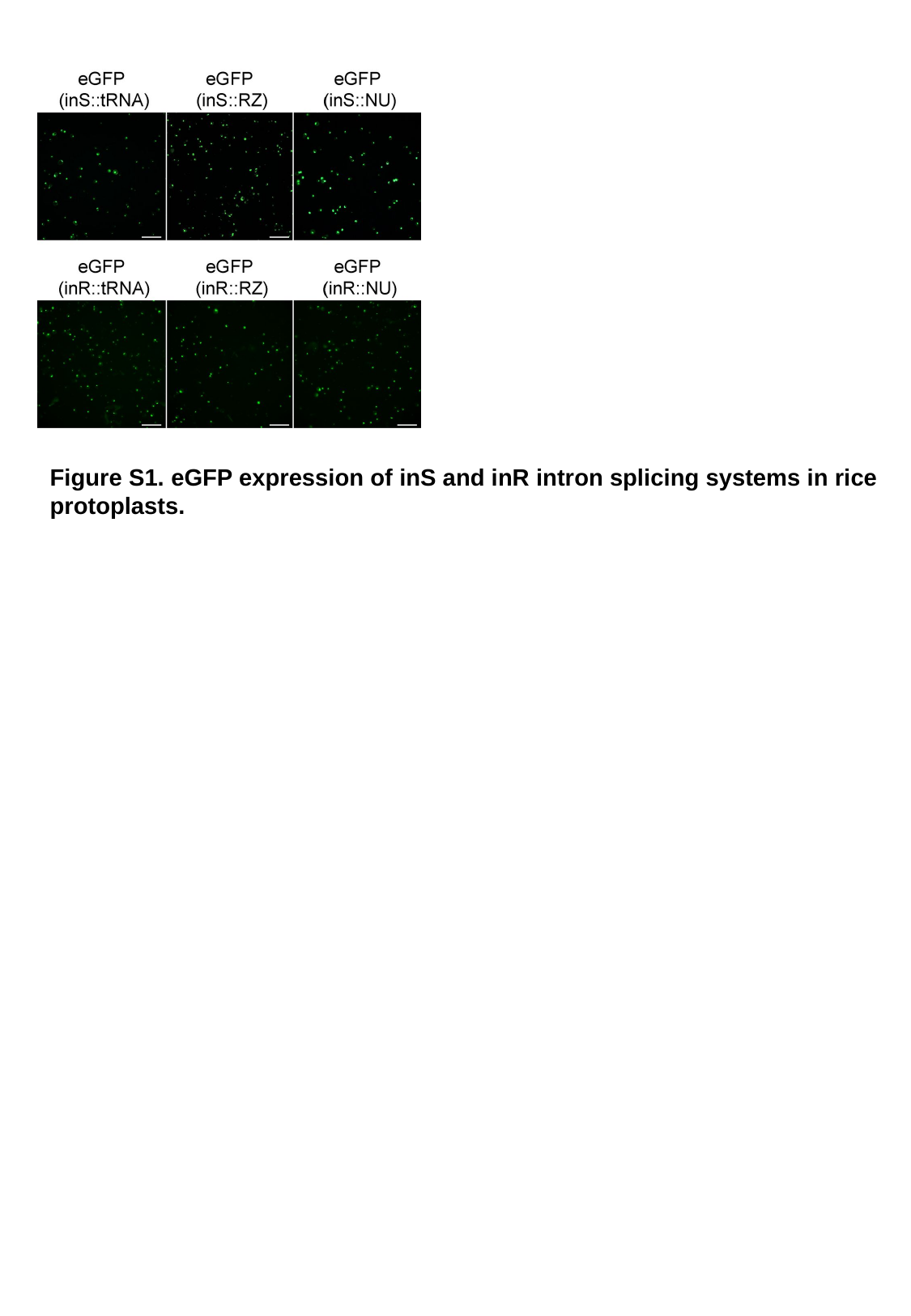

Figure S1. eGFP expression of inS and inR intron splicing systems in rice protoplasts.

## Slide 2
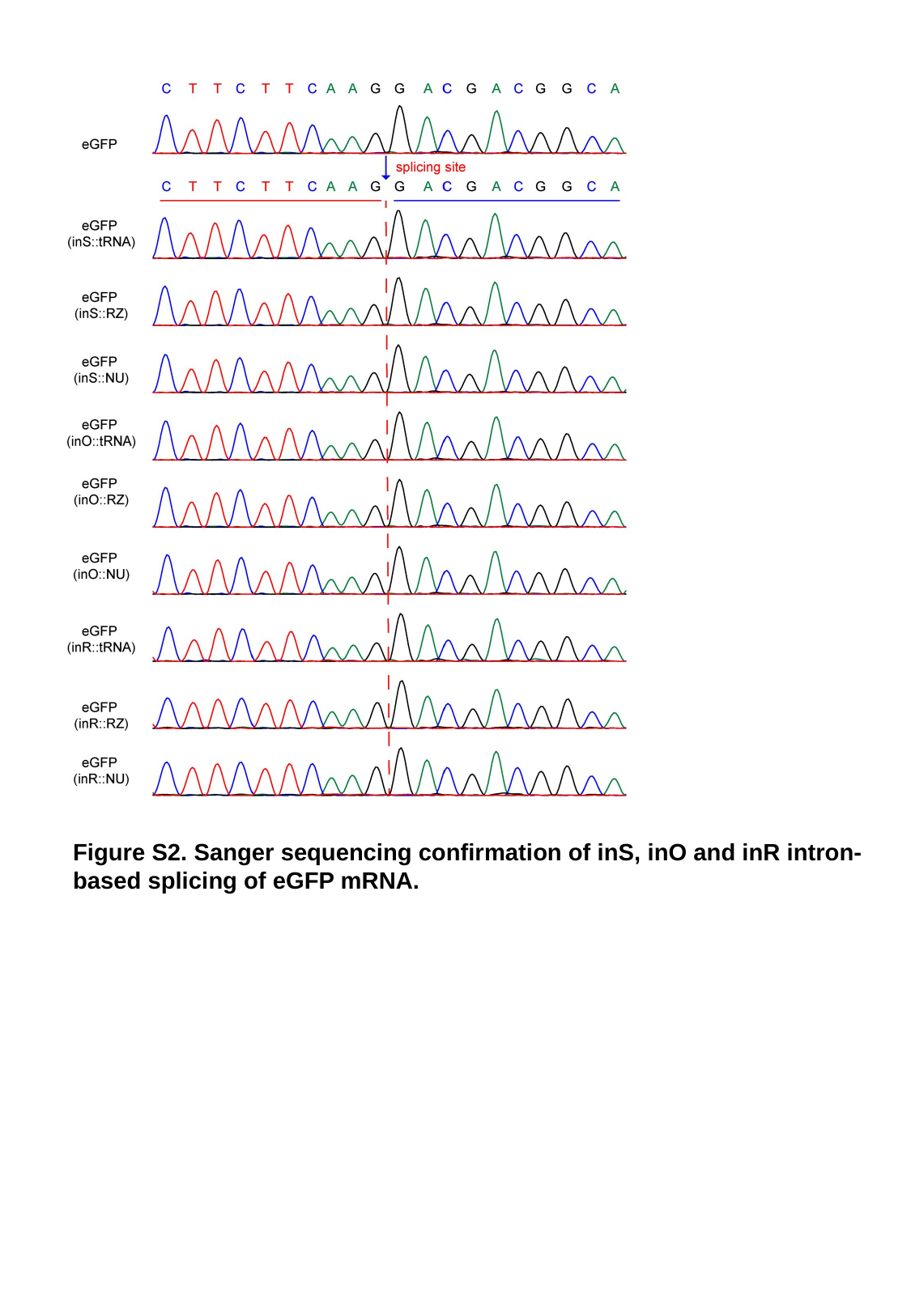

Figure S2. Sanger sequencing confirmation of inS, inO and inR intron-based splicing of eGFP mRNA.

## Slide 3
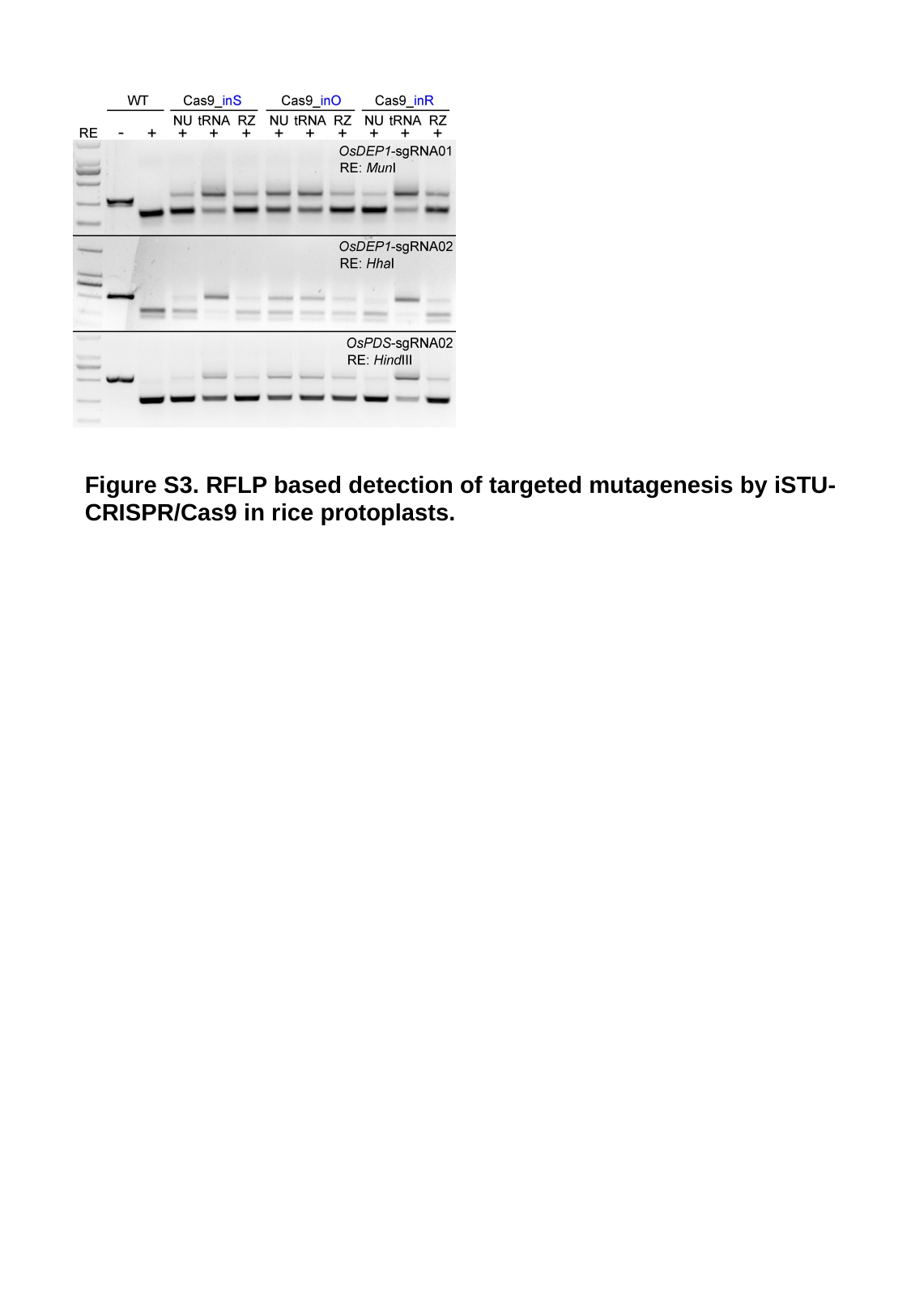

Figure S3. RFLP based detection of targeted mutagenesis by iSTU-CRISPR/Cas9 in rice protoplasts.

## Slide 4
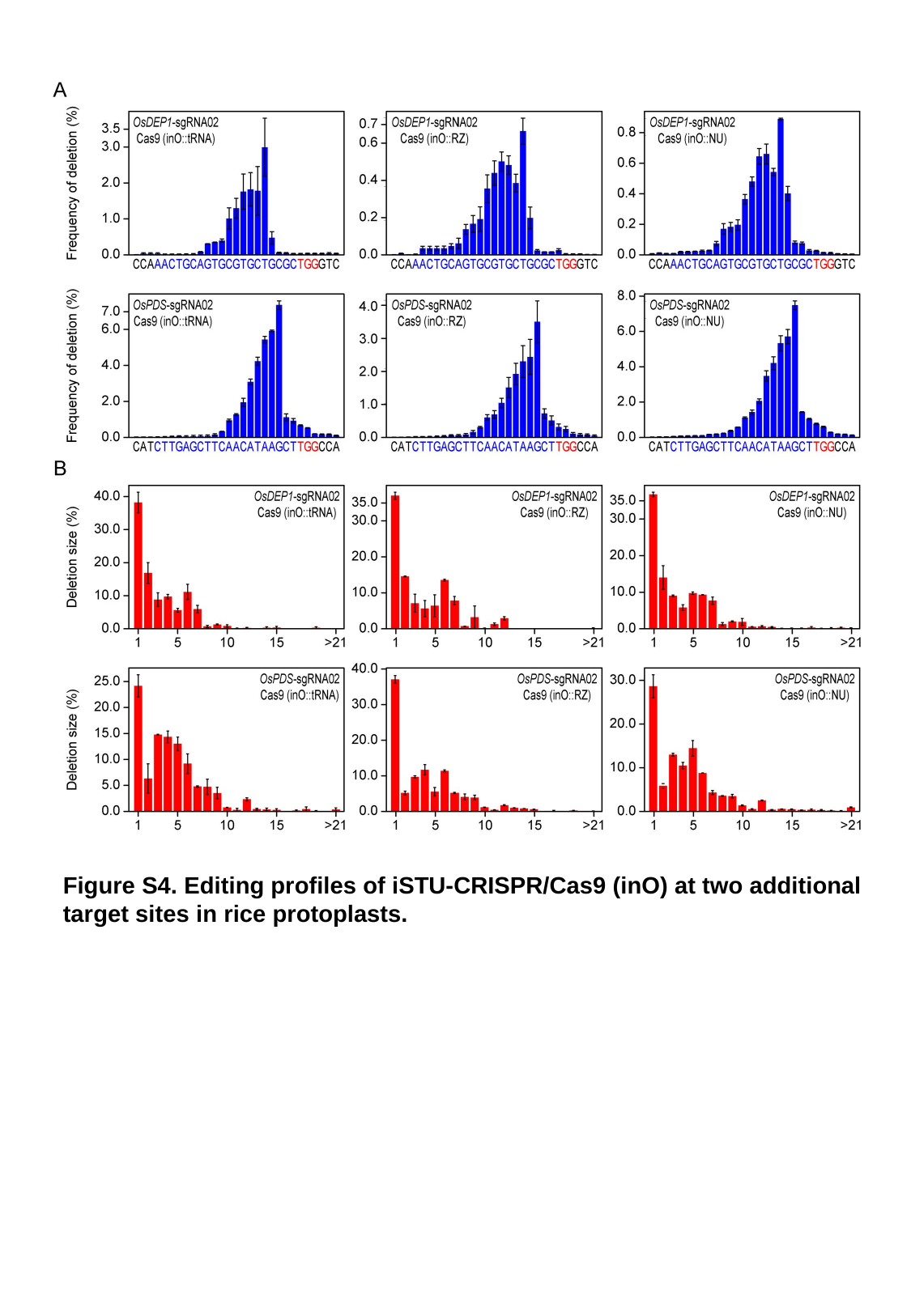

Figure S4. Editing profiles of iSTU-CRISPR/Cas9 (inO) at two additional target sites in rice protoplasts.

## Slide 5
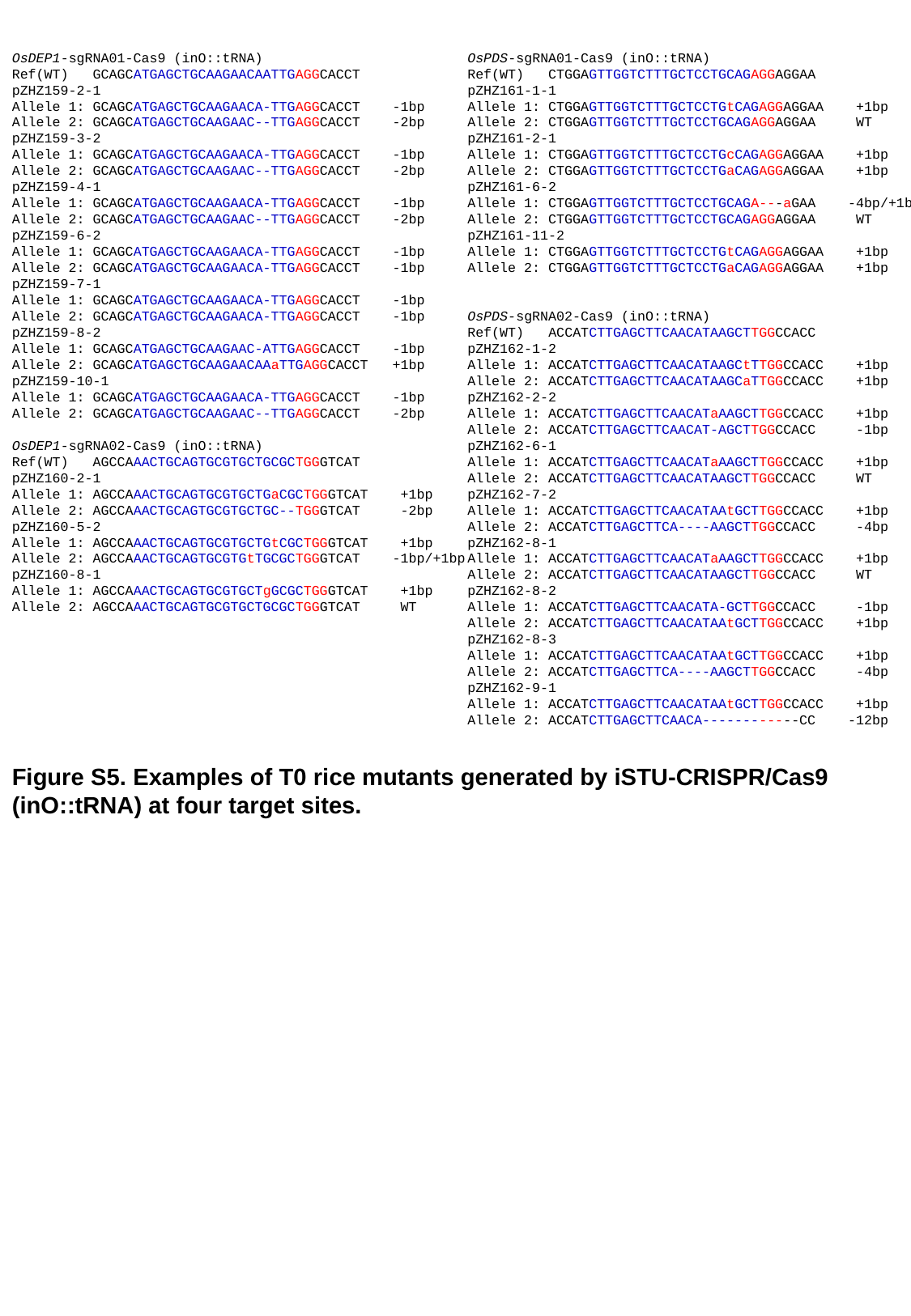

OsDEP1-sgRNA01-Cas9 (inO::tRNA)
Ref(WT) GCAGCATGAGCTGCAAGAACAATTGAGGCACCT
pZHZ159-2-1
Allele 1: GCAGCATGAGCTGCAAGAACA-TTGAGGCACCT -1bp
Allele 2: GCAGCATGAGCTGCAAGAAC--TTGAGGCACCT -2bp
pZHZ159-3-2
Allele 1: GCAGCATGAGCTGCAAGAACA-TTGAGGCACCT -1bp
Allele 2: GCAGCATGAGCTGCAAGAAC--TTGAGGCACCT -2bp
pZHZ159-4-1
Allele 1: GCAGCATGAGCTGCAAGAACA-TTGAGGCACCT -1bp
Allele 2: GCAGCATGAGCTGCAAGAAC--TTGAGGCACCT -2bp
pZHZ159-6-2
Allele 1: GCAGCATGAGCTGCAAGAACA-TTGAGGCACCT -1bp
Allele 2: GCAGCATGAGCTGCAAGAACA-TTGAGGCACCT -1bp
pZHZ159-7-1
Allele 1: GCAGCATGAGCTGCAAGAACA-TTGAGGCACCT -1bp
Allele 2: GCAGCATGAGCTGCAAGAACA-TTGAGGCACCT -1bp
pZHZ159-8-2
Allele 1: GCAGCATGAGCTGCAAGAAC-ATTGAGGCACCT -1bp
Allele 2: GCAGCATGAGCTGCAAGAACAAaTTGAGGCACCT +1bp
pZHZ159-10-1
Allele 1: GCAGCATGAGCTGCAAGAACA-TTGAGGCACCT -1bp
Allele 2: GCAGCATGAGCTGCAAGAAC--TTGAGGCACCT -2bp
OsDEP1-sgRNA02-Cas9 (inO::tRNA)
Ref(WT) AGCCAAACTGCAGTGCGTGCTGCGCTGGGTCAT
pZHZ160-2-1
Allele 1: AGCCAAACTGCAGTGCGTGCTGaCGCTGGGTCAT +1bp
Allele 2: AGCCAAACTGCAGTGCGTGCTGC--TGGGTCAT -2bp
pZHZ160-5-2
Allele 1: AGCCAAACTGCAGTGCGTGCTGtCGCTGGGTCAT +1bp
Allele 2: AGCCAAACTGCAGTGCGTGtTGCGCTGGGTCAT -1bp/+1bp
pZHZ160-8-1
Allele 1: AGCCAAACTGCAGTGCGTGCTgGCGCTGGGTCAT +1bp
Allele 2: AGCCAAACTGCAGTGCGTGCTGCGCTGGGTCAT WT
OsPDS-sgRNA01-Cas9 (inO::tRNA)
Ref(WT) CTGGAGTTGGTCTTTGCTCCTGCAGAGGAGGAA
pZHZ161-1-1
Allele 1: CTGGAGTTGGTCTTTGCTCCTGtCAGAGGAGGAA +1bp
Allele 2: CTGGAGTTGGTCTTTGCTCCTGCAGAGGAGGAA WT
pZHZ161-2-1
Allele 1: CTGGAGTTGGTCTTTGCTCCTGcCAGAGGAGGAA +1bp
Allele 2: CTGGAGTTGGTCTTTGCTCCTGaCAGAGGAGGAA +1bp
pZHZ161-6-2
Allele 1: CTGGAGTTGGTCTTTGCTCCTGCAGA---aGAA -4bp/+1bp
Allele 2: CTGGAGTTGGTCTTTGCTCCTGCAGAGGAGGAA WT
pZHZ161-11-2
Allele 1: CTGGAGTTGGTCTTTGCTCCTGtCAGAGGAGGAA +1bp
Allele 2: CTGGAGTTGGTCTTTGCTCCTGaCAGAGGAGGAA +1bp
OsPDS-sgRNA02-Cas9 (inO::tRNA)
Ref(WT) ACCATCTTGAGCTTCAACATAAGCTTGGCCACC
pZHZ162-1-2
Allele 1: ACCATCTTGAGCTTCAACATAAGCtTTGGCCACC +1bp
Allele 2: ACCATCTTGAGCTTCAACATAAGCaTTGGCCACC +1bp
pZHZ162-2-2
Allele 1: ACCATCTTGAGCTTCAACATaAAGCTTGGCCACC +1bp
Allele 2: ACCATCTTGAGCTTCAACAT-AGCTTGGCCACC -1bp
pZHZ162-6-1
Allele 1: ACCATCTTGAGCTTCAACATaAAGCTTGGCCACC +1bp
Allele 2: ACCATCTTGAGCTTCAACATAAGCTTGGCCACC WT
pZHZ162-7-2
Allele 1: ACCATCTTGAGCTTCAACATAAtGCTTGGCCACC +1bp
Allele 2: ACCATCTTGAGCTTCA----AAGCTTGGCCACC -4bp
pZHZ162-8-1
Allele 1: ACCATCTTGAGCTTCAACATaAAGCTTGGCCACC +1bp
Allele 2: ACCATCTTGAGCTTCAACATAAGCTTGGCCACC WT
pZHZ162-8-2
Allele 1: ACCATCTTGAGCTTCAACATA-GCTTGGCCACC -1bp
Allele 2: ACCATCTTGAGCTTCAACATAAtGCTTGGCCACC +1bp
pZHZ162-8-3
Allele 1: ACCATCTTGAGCTTCAACATAAtGCTTGGCCACC +1bp
Allele 2: ACCATCTTGAGCTTCA----AAGCTTGGCCACC -4bp
pZHZ162-9-1
Allele 1: ACCATCTTGAGCTTCAACATAAtGCTTGGCCACC +1bp
Allele 2: ACCATCTTGAGCTTCAACA------------CC -12bp
Figure S5. Examples of T0 rice mutants generated by iSTU-CRISPR/Cas9 (inO::tRNA) at four target sites.

## Slide 6
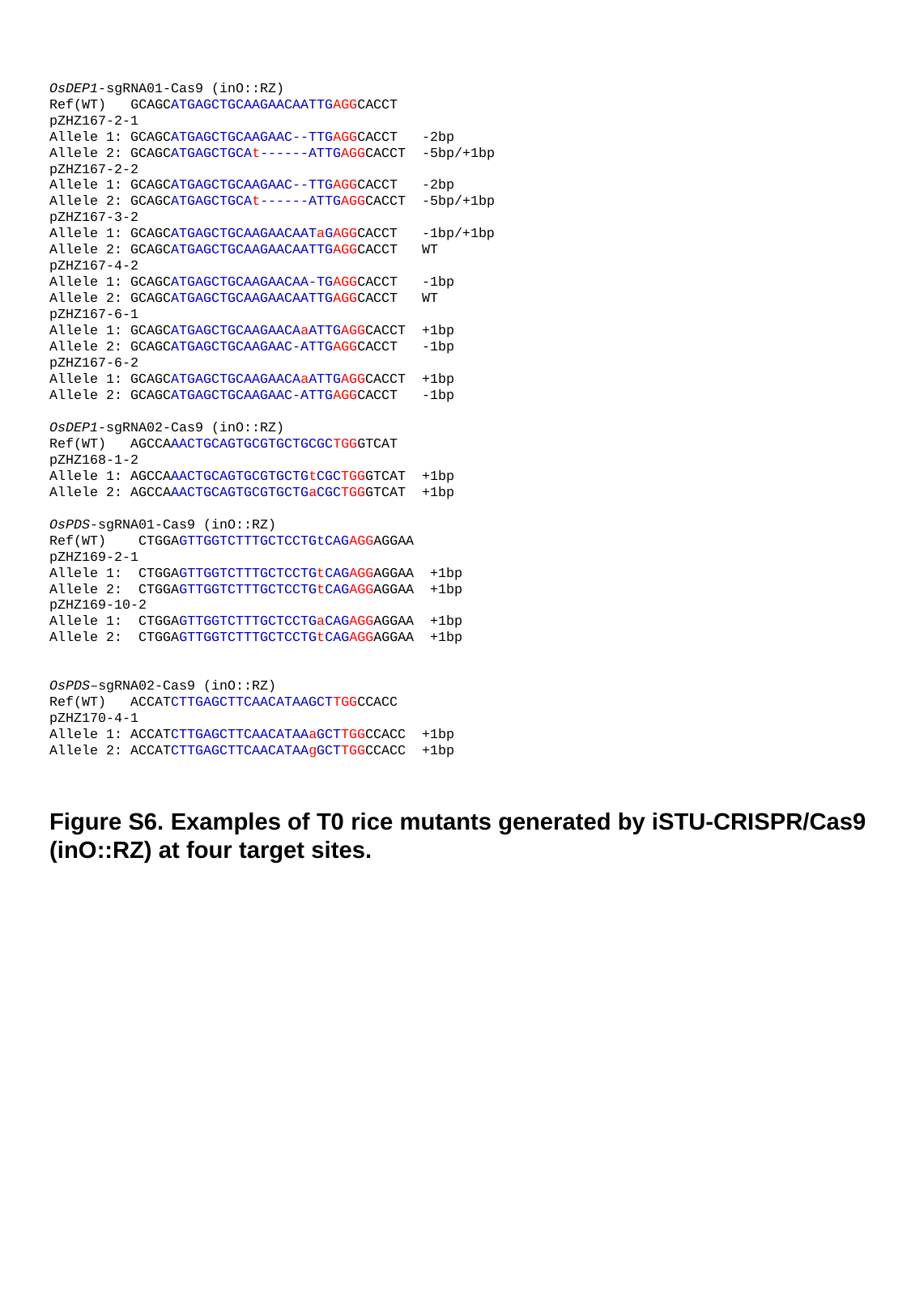

OsDEP1-sgRNA01-Cas9 (inO::RZ)
Ref(WT) GCAGCATGAGCTGCAAGAACAATTGAGGCACCT
pZHZ167-2-1
Allele 1: GCAGCATGAGCTGCAAGAAC--TTGAGGCACCT -2bp
Allele 2: GCAGCATGAGCTGCAt------ATTGAGGCACCT -5bp/+1bp
pZHZ167-2-2
Allele 1: GCAGCATGAGCTGCAAGAAC--TTGAGGCACCT -2bp
Allele 2: GCAGCATGAGCTGCAt------ATTGAGGCACCT -5bp/+1bp
pZHZ167-3-2
Allele 1: GCAGCATGAGCTGCAAGAACAATaGAGGCACCT -1bp/+1bp
Allele 2: GCAGCATGAGCTGCAAGAACAATTGAGGCACCT WT
pZHZ167-4-2
Allele 1: GCAGCATGAGCTGCAAGAACAA-TGAGGCACCT -1bp
Allele 2: GCAGCATGAGCTGCAAGAACAATTGAGGCACCT WT
pZHZ167-6-1
Allele 1: GCAGCATGAGCTGCAAGAACAaATTGAGGCACCT +1bp
Allele 2: GCAGCATGAGCTGCAAGAAC-ATTGAGGCACCT -1bp
pZHZ167-6-2
Allele 1: GCAGCATGAGCTGCAAGAACAaATTGAGGCACCT +1bp
Allele 2: GCAGCATGAGCTGCAAGAAC-ATTGAGGCACCT -1bp
OsDEP1-sgRNA02-Cas9 (inO::RZ)
Ref(WT) AGCCAAACTGCAGTGCGTGCTGCGCTGGGTCAT
pZHZ168-1-2
Allele 1: AGCCAAACTGCAGTGCGTGCTGtCGCTGGGTCAT +1bp
Allele 2: AGCCAAACTGCAGTGCGTGCTGaCGCTGGGTCAT +1bp
OsPDS-sgRNA01-Cas9 (inO::RZ)
Ref(WT) CTGGAGTTGGTCTTTGCTCCTGtCAGAGGAGGAA
pZHZ169-2-1
Allele 1: CTGGAGTTGGTCTTTGCTCCTGtCAGAGGAGGAA +1bp
Allele 2: CTGGAGTTGGTCTTTGCTCCTGtCAGAGGAGGAA +1bp
pZHZ169-10-2
Allele 1: CTGGAGTTGGTCTTTGCTCCTGaCAGAGGAGGAA +1bp
Allele 2: CTGGAGTTGGTCTTTGCTCCTGtCAGAGGAGGAA +1bp
OsPDS–sgRNA02-Cas9 (inO::RZ)
Ref(WT) ACCATCTTGAGCTTCAACATAAGCTTGGCCACC
pZHZ170-4-1
Allele 1: ACCATCTTGAGCTTCAACATAAaGCTTGGCCACC +1bp
Allele 2: ACCATCTTGAGCTTCAACATAAgGCTTGGCCACC +1bp
Figure S6. Examples of T0 rice mutants generated by iSTU-CRISPR/Cas9 (inO::RZ) at four target sites.

## Slide 7
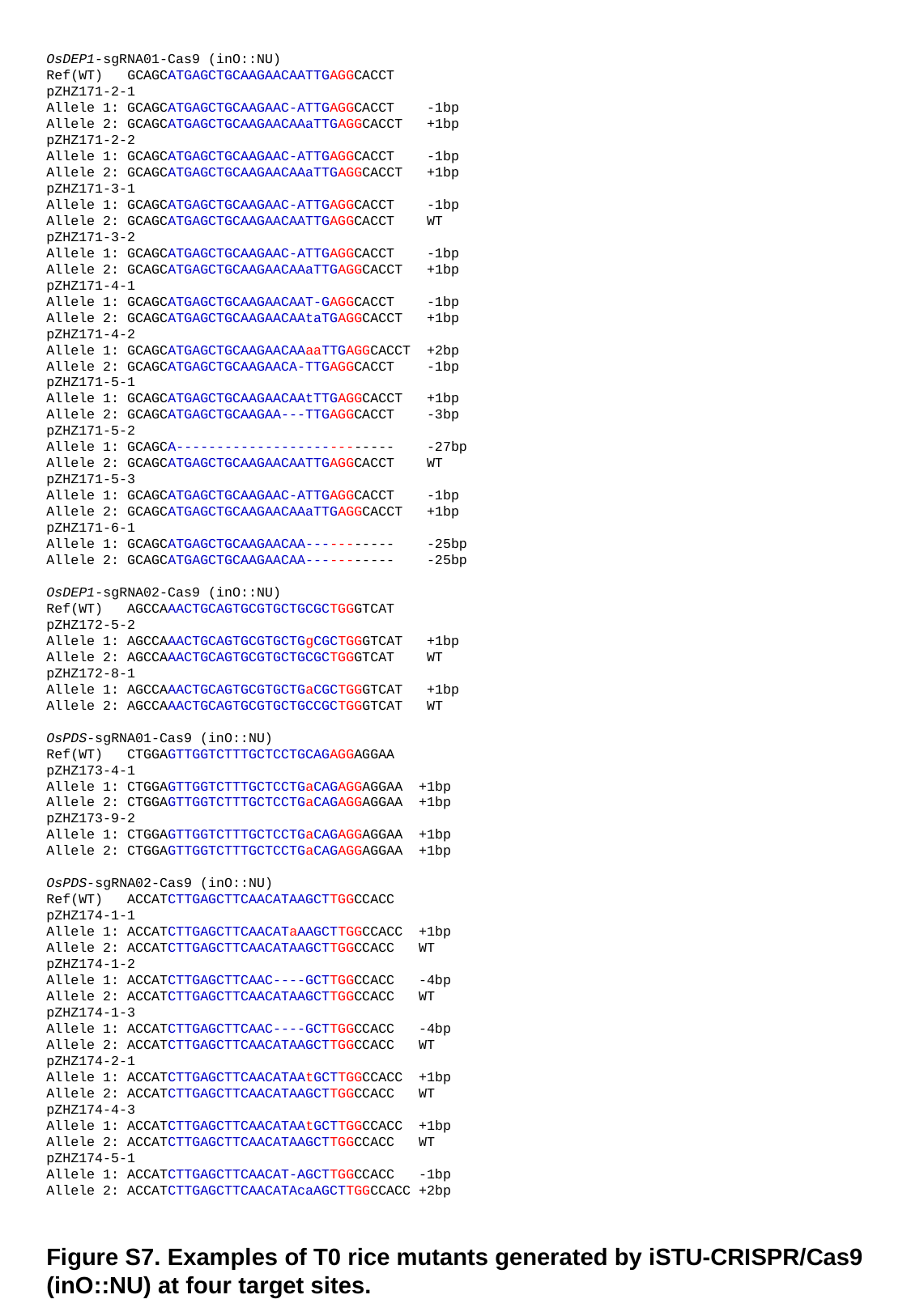

OsDEP1-sgRNA01-Cas9 (inO::NU)
Ref(WT) GCAGCATGAGCTGCAAGAACAATTGAGGCACCT
pZHZ171-2-1
Allele 1: GCAGCATGAGCTGCAAGAAC-ATTGAGGCACCT -1bp
Allele 2: GCAGCATGAGCTGCAAGAACAAaTTGAGGCACCT +1bp
pZHZ171-2-2
Allele 1: GCAGCATGAGCTGCAAGAAC-ATTGAGGCACCT -1bp
Allele 2: GCAGCATGAGCTGCAAGAACAAaTTGAGGCACCT +1bp
pZHZ171-3-1
Allele 1: GCAGCATGAGCTGCAAGAAC-ATTGAGGCACCT -1bp
Allele 2: GCAGCATGAGCTGCAAGAACAATTGAGGCACCT WT
pZHZ171-3-2
Allele 1: GCAGCATGAGCTGCAAGAAC-ATTGAGGCACCT -1bp
Allele 2: GCAGCATGAGCTGCAAGAACAAaTTGAGGCACCT +1bp
pZHZ171-4-1
Allele 1: GCAGCATGAGCTGCAAGAACAAT-GAGGCACCT -1bp
Allele 2: GCAGCATGAGCTGCAAGAACAAtaTGAGGCACCT +1bp
pZHZ171-4-2
Allele 1: GCAGCATGAGCTGCAAGAACAAaaTTGAGGCACCT +2bp
Allele 2: GCAGCATGAGCTGCAAGAACA-TTGAGGCACCT -1bp
pZHZ171-5-1
Allele 1: GCAGCATGAGCTGCAAGAACAAtTTGAGGCACCT +1bp
Allele 2: GCAGCATGAGCTGCAAGAA---TTGAGGCACCT -3bp
pZHZ171-5-2
Allele 1: GCAGCA--------------------------- -27bp
Allele 2: GCAGCATGAGCTGCAAGAACAATTGAGGCACCT WT
pZHZ171-5-3
Allele 1: GCAGCATGAGCTGCAAGAAC-ATTGAGGCACCT -1bp
Allele 2: GCAGCATGAGCTGCAAGAACAAaTTGAGGCACCT +1bp
pZHZ171-6-1
Allele 1: GCAGCATGAGCTGCAAGAACAA----------- -25bp
Allele 2: GCAGCATGAGCTGCAAGAACAA----------- -25bp
OsDEP1-sgRNA02-Cas9 (inO::NU)
Ref(WT) AGCCAAACTGCAGTGCGTGCTGCGCTGGGTCAT
pZHZ172-5-2
Allele 1: AGCCAAACTGCAGTGCGTGCTGgCGCTGGGTCAT +1bp
Allele 2: AGCCAAACTGCAGTGCGTGCTGCGCTGGGTCAT WT
pZHZ172-8-1
Allele 1: AGCCAAACTGCAGTGCGTGCTGaCGCTGGGTCAT +1bp
Allele 2: AGCCAAACTGCAGTGCGTGCTGCCGCTGGGTCAT WT
OsPDS-sgRNA01-Cas9 (inO::NU)
Ref(WT) CTGGAGTTGGTCTTTGCTCCTGCAGAGGAGGAA
pZHZ173-4-1
Allele 1: CTGGAGTTGGTCTTTGCTCCTGaCAGAGGAGGAA +1bp
Allele 2: CTGGAGTTGGTCTTTGCTCCTGaCAGAGGAGGAA +1bp
pZHZ173-9-2
Allele 1: CTGGAGTTGGTCTTTGCTCCTGaCAGAGGAGGAA +1bp
Allele 2: CTGGAGTTGGTCTTTGCTCCTGaCAGAGGAGGAA +1bp
OsPDS-sgRNA02-Cas9 (inO::NU)
Ref(WT) ACCATCTTGAGCTTCAACATAAGCTTGGCCACC
pZHZ174-1-1
Allele 1: ACCATCTTGAGCTTCAACATaAAGCTTGGCCACC +1bp
Allele 2: ACCATCTTGAGCTTCAACATAAGCTTGGCCACC WT
pZHZ174-1-2
Allele 1: ACCATCTTGAGCTTCAAC----GCTTGGCCACC -4bp
Allele 2: ACCATCTTGAGCTTCAACATAAGCTTGGCCACC WT
pZHZ174-1-3
Allele 1: ACCATCTTGAGCTTCAAC----GCTTGGCCACC -4bp
Allele 2: ACCATCTTGAGCTTCAACATAAGCTTGGCCACC WT
pZHZ174-2-1
Allele 1: ACCATCTTGAGCTTCAACATAAtGCTTGGCCACC +1bp
Allele 2: ACCATCTTGAGCTTCAACATAAGCTTGGCCACC WT
pZHZ174-4-3
Allele 1: ACCATCTTGAGCTTCAACATAAtGCTTGGCCACC +1bp
Allele 2: ACCATCTTGAGCTTCAACATAAGCTTGGCCACC WT
pZHZ174-5-1
Allele 1: ACCATCTTGAGCTTCAACAT-AGCTTGGCCACC -1bp
Allele 2: ACCATCTTGAGCTTCAACATAcaAGCTTGGCCACC +2bp
Figure S7. Examples of T0 rice mutants generated by iSTU-CRISPR/Cas9 (inO::NU) at four target sites.

## Slide 8
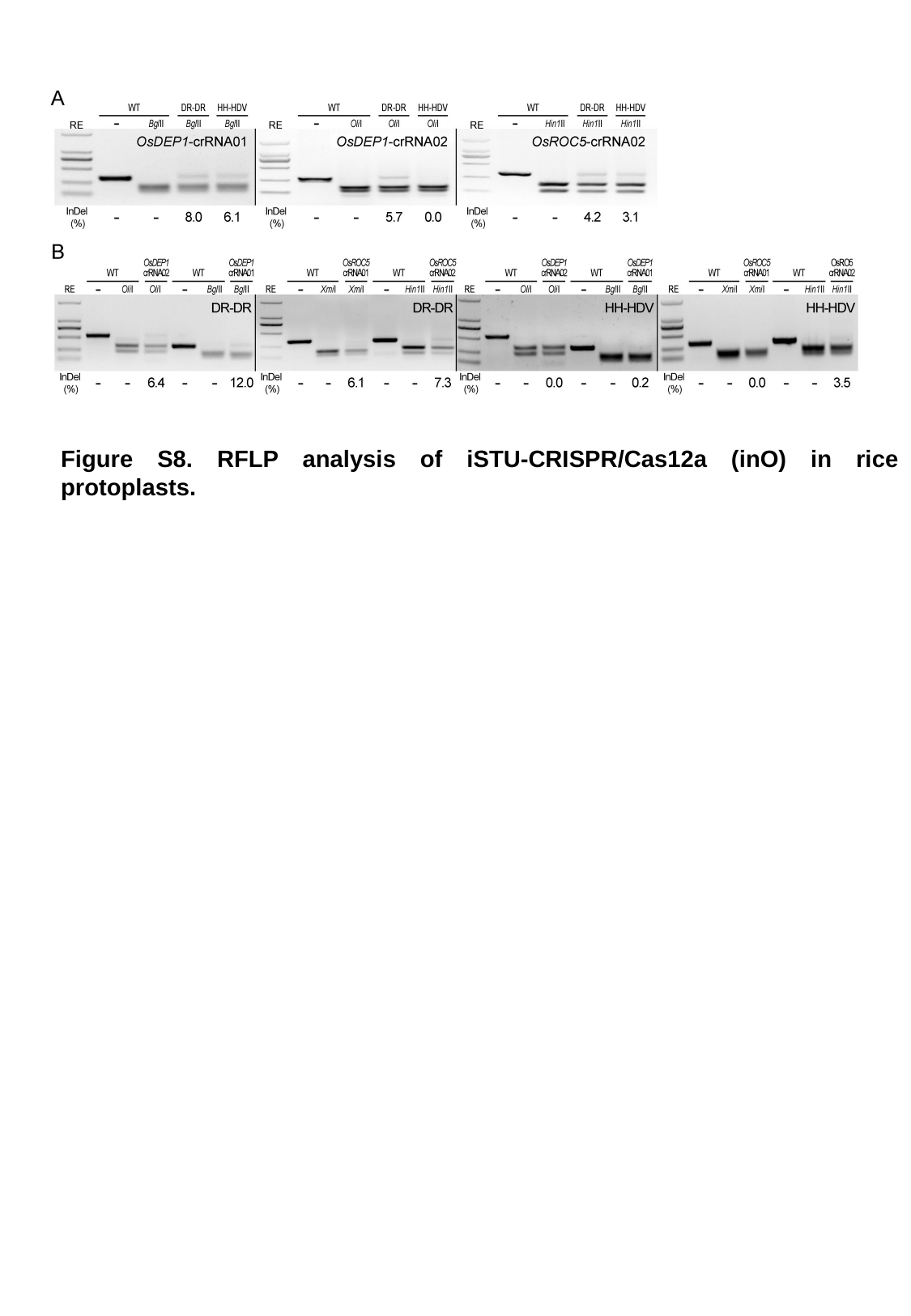

Figure S8. RFLP analysis of iSTU-CRISPR/Cas12a (inO) in rice protoplasts.

## Slide 9
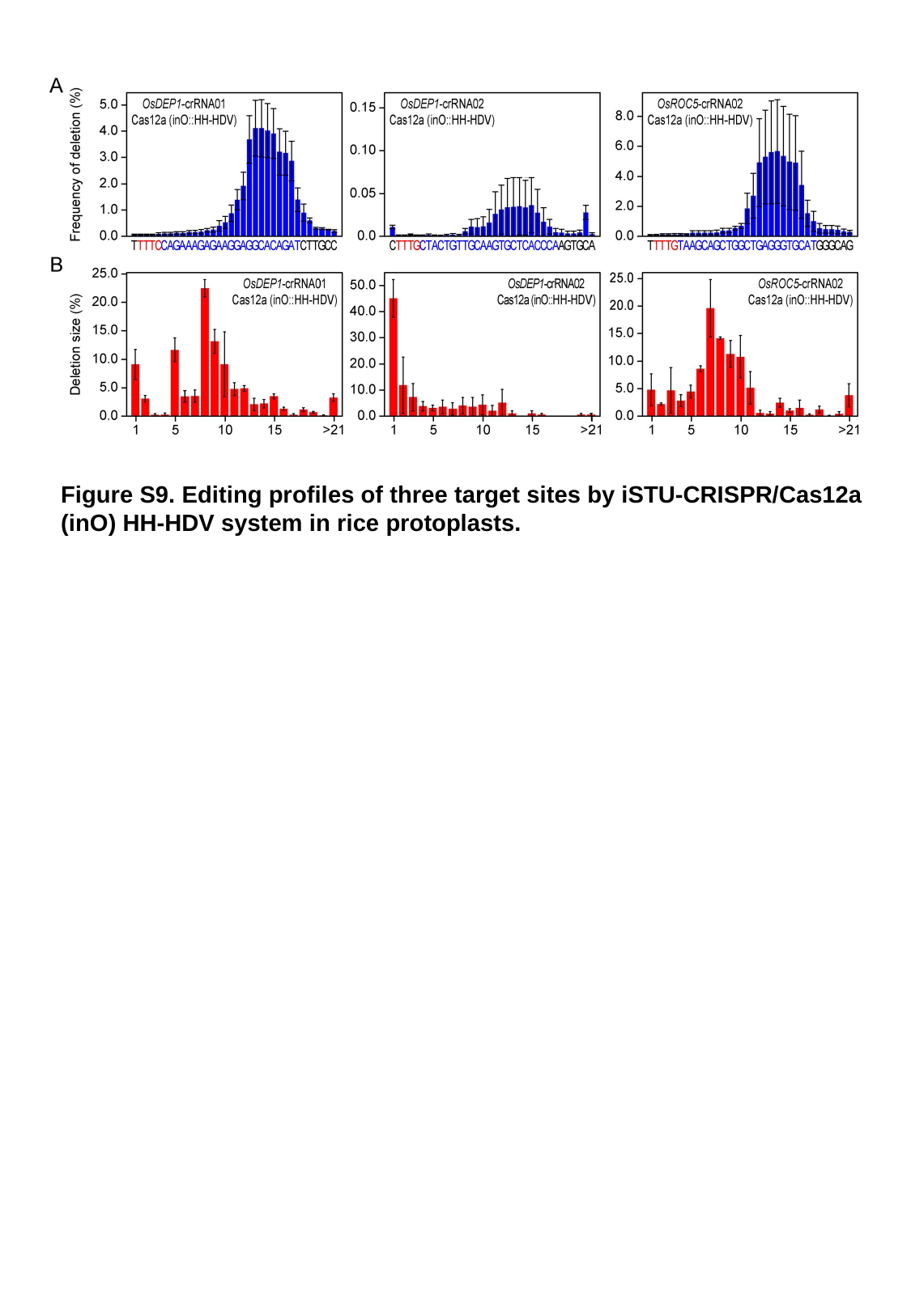

Figure S9. Editing profiles of three target sites by iSTU-CRISPR/Cas12a (inO) HH-HDV system in rice protoplasts.

## Slide 10
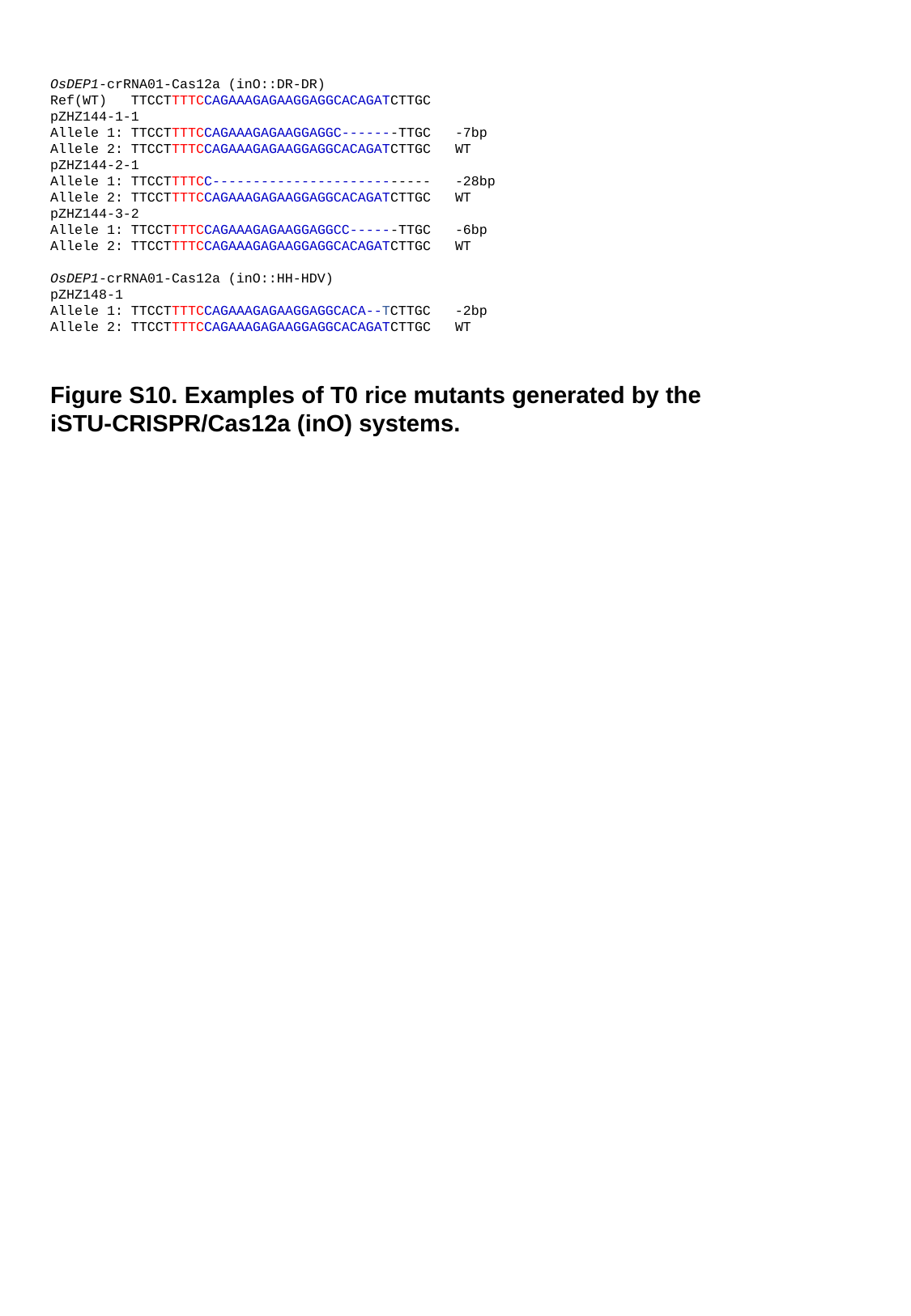

OsDEP1-crRNA01-Cas12a (inO::DR-DR)
Ref(WT) TTCCTTTTCCAGAAAGAGAAGGAGGCACAGATCTTGC
pZHZ144-1-1
Allele 1: TTCCTTTTCCAGAAAGAGAAGGAGGC-------TTGC -7bp
Allele 2: TTCCTTTTCCAGAAAGAGAAGGAGGCACAGATCTTGC WT
pZHZ144-2-1
Allele 1: TTCCTTTTCC--------------------------- -28bp
Allele 2: TTCCTTTTCCAGAAAGAGAAGGAGGCACAGATCTTGC WT
pZHZ144-3-2
Allele 1: TTCCTTTTCCAGAAAGAGAAGGAGGCC------TTGC -6bp
Allele 2: TTCCTTTTCCAGAAAGAGAAGGAGGCACAGATCTTGC WT
OsDEP1-crRNA01-Cas12a (inO::HH-HDV)
pZHZ148-1
Allele 1: TTCCTTTTCCAGAAAGAGAAGGAGGCACA--TCTTGC -2bp
Allele 2: TTCCTTTTCCAGAAAGAGAAGGAGGCACAGATCTTGC WT
Figure S10. Examples of T0 rice mutants generated by the iSTU-CRISPR/Cas12a (inO) systems.
